# The role of HER2 and HER3 in HER2-amplified cancers beyond breast cancers

**DOI:** 10.1038/s41598-021-88683-w

**Published:** 2021-04-27

**Authors:** Avisek Majumder, Manbir Sandhu, Debarko Banerji, Veronica Steri, Adam Olshen, Mark M. Moasser

**Affiliations:** 1 Department of Medicine, University of California, San Francisco, Box 3111, San Francisco, CA 94143 USA; 2 Department of Structural Biology, St. Jude Children’s Research Hospital, 262 Danny Thomas Place, Memphis, TN 38105-3678 USA; 3 Genentech, Inc, 1 DNA Way, South San Francisco, CA 94080-4990 USA; 4 Helen Diller Family Comprehensive Cancer Center, University of California, San Francisco, San Francisco, CA 94143 USA; 5 Department of Epidemiology and Biostatistics, University of California, San Francisco, San Francisco, CA 94143 USA

**Keywords:** Cancer, Oncogenes

## Abstract

HER2 and HER3 play key driving functions in the pathophysiology of HER2-amplified breast cancers, but this function is less well characterized in other cancers driven by HER2 amplification. This study aimed to explore the role of HER2 and HER3 signaling in other types of HER2-amplified cancer. The expression and signaling activity of HER2, HER3, and downstream pathway proteins were studied in cell panels representing HER2-amplified cancers of the breast, bladder, colon and rectal, stomach, esophagus, lung, tongue, and endometrium along with controls lacking HER2 amplification. We report that HER2-amplified cancers are addicted to HER2 across different cancer types and the depth of addiction is best linked with the expression level of HER2, but not with HER3 expression. We report that the expression and constitutive phosphorylation of HER3 are ubiquitous in HER2-amplified breast cancer cell lines, but much more variable in HER2-amplified cancer cells from other tissues. We observed the lapatinib-induced compensatory upregulation of HER3 signaling in many types of HER2-amplified cancers, although with much variability. We find that HER3 expression is essential for in vivo tumorigenic growth in some HER2-amplified tumors but not others. Importantly HER3 expression level does not correlate well with its functional importance. More biomarkers will be needed to guide the optimal use of HER3 inhibitors in HER2-amplified cancers from non-breast origin. Unlike oncogenes activated through mutational events, the activation of HER2 through overexpression represents a gradient of activities and depth of addiction and the response to inhibitors follows a similar gradient.

## Introduction

About 20% of breast cancers are driven by amplification of the ERBB2 gene and overexpression of its gene product, the HER2 protein. A large body of in vitro and in vivo experimental evidence supports the notion that these cancers are driven by overactive and constitutive HER2 signaling^1,2^. This understanding has fueled extensive efforts to develop treatments that target the HER2 oncoprotein with several agents already approved for use including antibodies, antibody–drug conjugates (ADCs), and small molecule kinase inhibitors^3,4^. The experience to date with HER2-targeting agents has revealed a degree of complexity with this amplified oncogene that sets it apart from oncogenes activated through gene mutation or fusion events such as EGFR or Alk in lung cancers, Bcr-abl in leukemias, or BRAF in melanomas. In particular, HER2 has proven to be a more effective target for chemodelivery or immunostimulatory agents such as antibodies or ADCs but a less effective target for kinase inhibitors.

Signaling in the HER family is mediated through receptor dimerization including homodimerization and heterodimerization^5^. The signaling functions of HER2 are closely linked with its HER family partner HER3 and this heterodimer complex forms one of the more favored and potent signaling dimers in this family^6^. The close relationship between HER2 and HER3 extends into the realm of oncogenic signaling^7^. Overexpression of HER2 or its mouse homolog Neu induce tumorigenesis when expressed in mouse mammary tissue, however these tumors fail to form if the HER3 gene is knocked out in these tissues^8^. Similarly, the transcriptional or shRNA knockdown of HER3 in HER2-amplified human cancer cell lines suppresses their in vivo tumorigenic growth^9,10^. These experimental studies have established that HER3 is an obligate partner in HER2-induced mammary tumorigenesis.

The role of HER3 in HER2-driven breast cancers extends beyond its role in the genesis and growth of these tumors and accounts for much of the observed resistance to HER2 inhibitors. Unlike HER2, the expression of HER3 is highly dynamic and highly regulated^11,12^. Its requisite function in HER2-amplified cancers is accomplished without significant overexpression. However HER3 is linked in a negative feedback circuit with downstream PI3K/Akt signaling and any attempts to suppress HER2-HER3 or downstream PI3K/Akt signaling using pharmacologic means induces a compensatory upregulation of HER3 within hours that functions to restore HER2-HER3 signaling output in the face of continued pharmacologic suppression^13–15^. This resiliency mitigates the efficacies of all HER2 inhibitors and underlies their modest clinical activities in the treatment of HER2-amplified breast cancers^16–19^. The HER3 kinase domain is catalytically inactive and it functions predominantly as an allosteric activator of the other HER family kinase domains, such as it does with HER2^20^. Its compensatory upregulation enables more efficient signaling in partnership with any residual HER2 kinase activity^13,14^. These findings have redefined the HER2-HER3 complex as the functionally relevant tumor driver in HER2-amplified tumors and established the bar for the development of therapies with much higher clinical efficacies. Efforts are underway to develop effective inhibitors of HER3 to combine with inhibitors of HER2 for much more effective treatment of HER2-amplified breast cancers.

The more recent genomic characterization of human cancers has revealed that the amplification of HER2 is seen in many types of epithelial cancers other than breast cancers, albeit at lower percentages. HER2 amplification is seen in cancers of the stomach and esophagus, colon, bladder, endometrium, salivary gland, lung, ovaries and other tissues^21–30^. The role of HER2 and HER3 in these other types of HER2-amplified cancers is less well defined than in breast cancers. Therefore, we set out to collect a panel of HER2-amplified cancer cell lines across tissue types to begin to determine whether the pathophysiology of HER2-amplified cancers is similar to breast cancers in other tumor types or whether there are significant differences. Some of the questions we asked were whether all HER2-amplified cancers are addicted to HER2, whether all HER2-amplified cancers express and require HER3, and whether they all exhibit the drug-induced compensatory upregulation of HER3. This undertaking involved the study of forty six cell lines in biological and signaling studies in vitro with three of them taken to xenograft models for in vivo studies.

## Results

**Figure 1:**
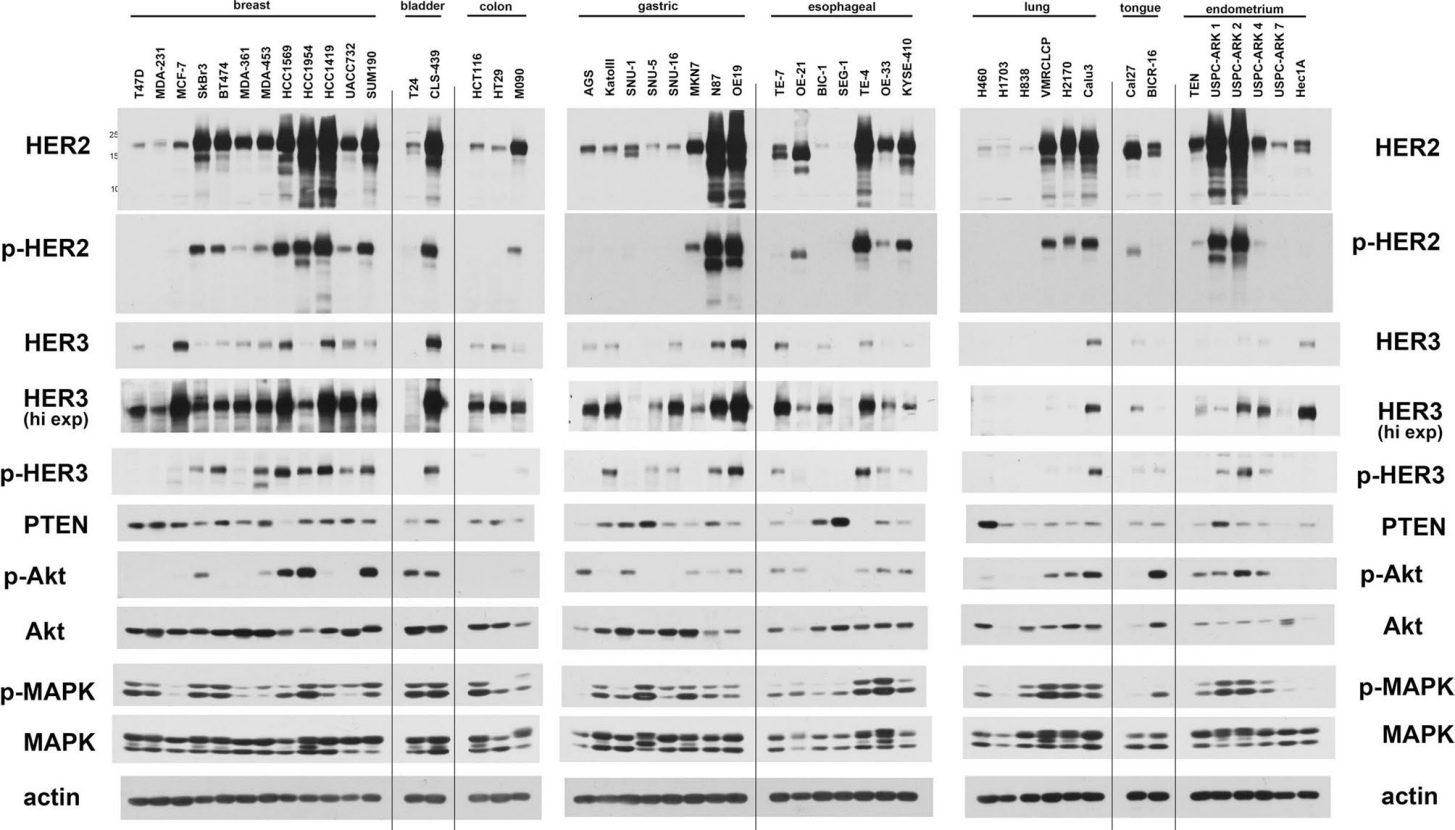
HER2/HER3 expression and signaling in a panel of cancer cells. Cell lysates from a panel of HER2-overexpressing cancer cell lines were immunoblotted as indicated. For each subtype of cancer, cell lines without HER2 overexpression were also used as comparators. The three gels were run and transferred simultaneously and the immunoblots were performed simultaneously using the same primary and secondary immunoblotting solution preparations.

**Figure 2:**
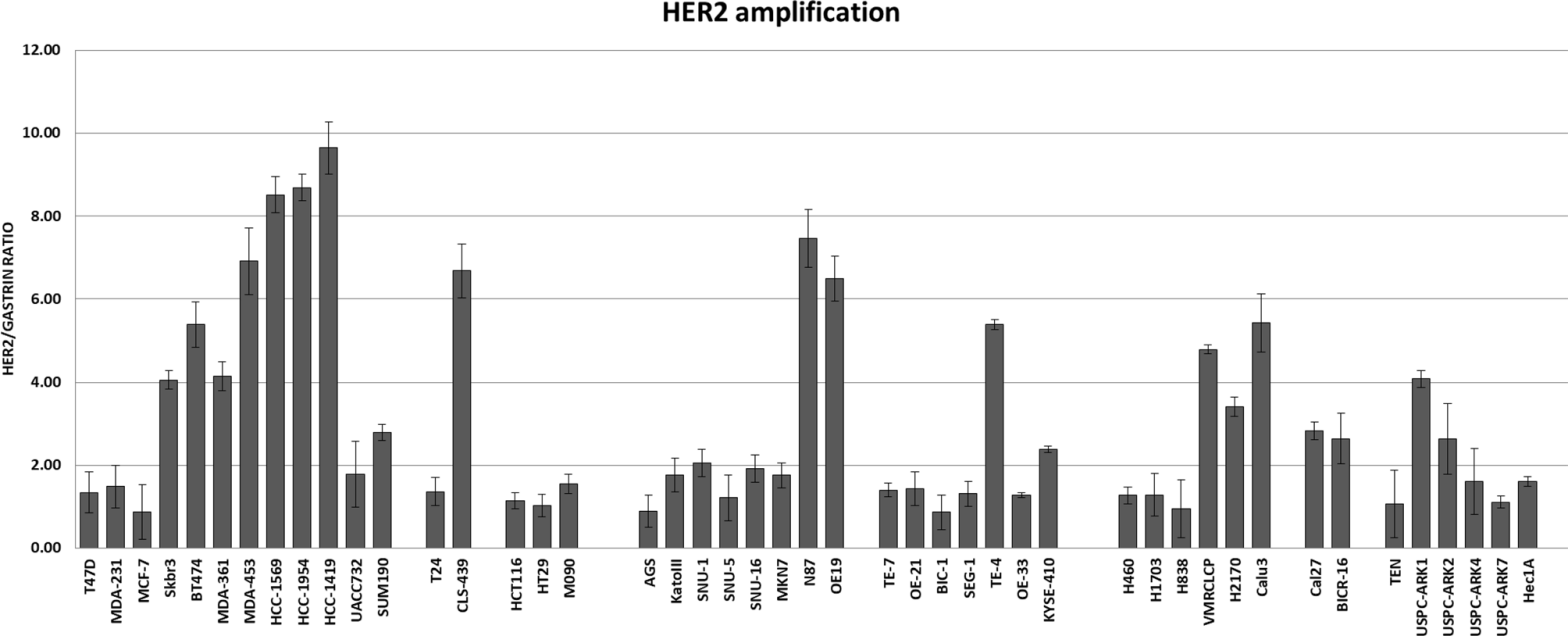
HER2 gene amplification in a panel of cancer cells. HER2 gene amplification status was assayed in the cell panel by qPCR using the gastrin gene as a chromosome 17 control. The data is shown as the average of triplicates with error bars indicating Std Dev.

We collected a panel of cancer cell lines with HER2 amplification from eight different tissue types (breast, bladder, colon, stomach, esophageal, lung, tongue, and endometrium). As a comparator for each tissue type, we also included one or more cancer cell lines without amplification of HER2. The relative expression and phosphorylation of HER2, HER3 and downstream signaling proteins were evaluated and compared by western blotting (Fig. 1). The relative amplification of the ERBB2 gene was assessed by qPCR (Fig. 2). The ERBB2 gene was quantified relative to the gastrin gene 2 MB upstream as control and thus functions as an imperfect surrogate for gene copy number that may not mirror copy number data available from other techniques. As expected, HER2 gene amplification correlates with HER2 protein expression across the cell panel. The expression of HER2 shows great variability among HER2-amplified cancer cell lines (Fig. 1). The expression of HER3 is ubiquitous in HER2-amplified breast cancer cell lines and while it is also expressed in many HER2-amplified cancer cells from other tissues, this expression varies considerably.

Some HER2-amplified cancer cells show very low HER3 protein expression (MKN7, VMRCLCP, USPC-ARK1, H2170). The constitutive phosphorylation of HER3 is seen predominantly in HER2-amplified cancer cells. However a few gastric and esophageal cancers without HER2-amplification show phosphorylation of HER3, suggesting the possibility of ligand-driven signaling or receptor cross-talk in these cancer cells (KatoIII, SNU-5, SNU-16, TE-7). Overall Akt and MAPK signaling showed a poor correlation with HER2 and HER3 signaling across the cell panel.

**Figure 3:**
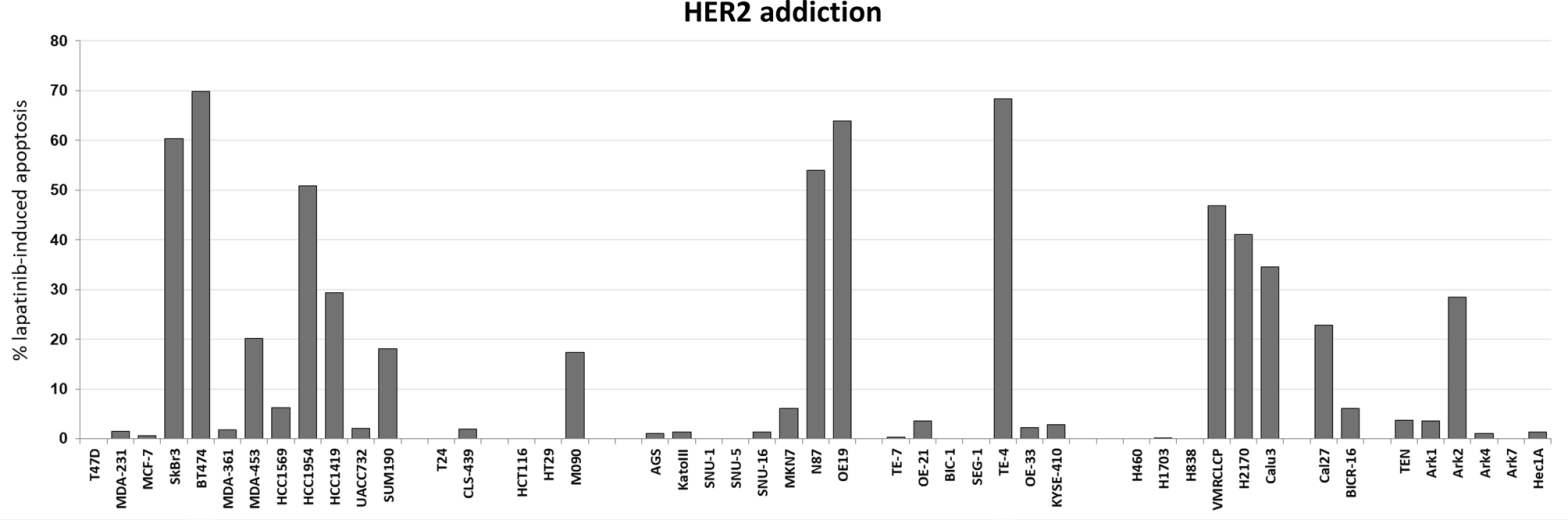
HER2 addiction in a panel of cancer cells. Cell lines were treated with 5uM lapatinib for 48–72 h and apoptotic cell death was assayed by flow cytometry analysis. The data plotted is the increase in % apoptosis over the DMSO control. The data shown here is from one experiment.

**Figure 4:**
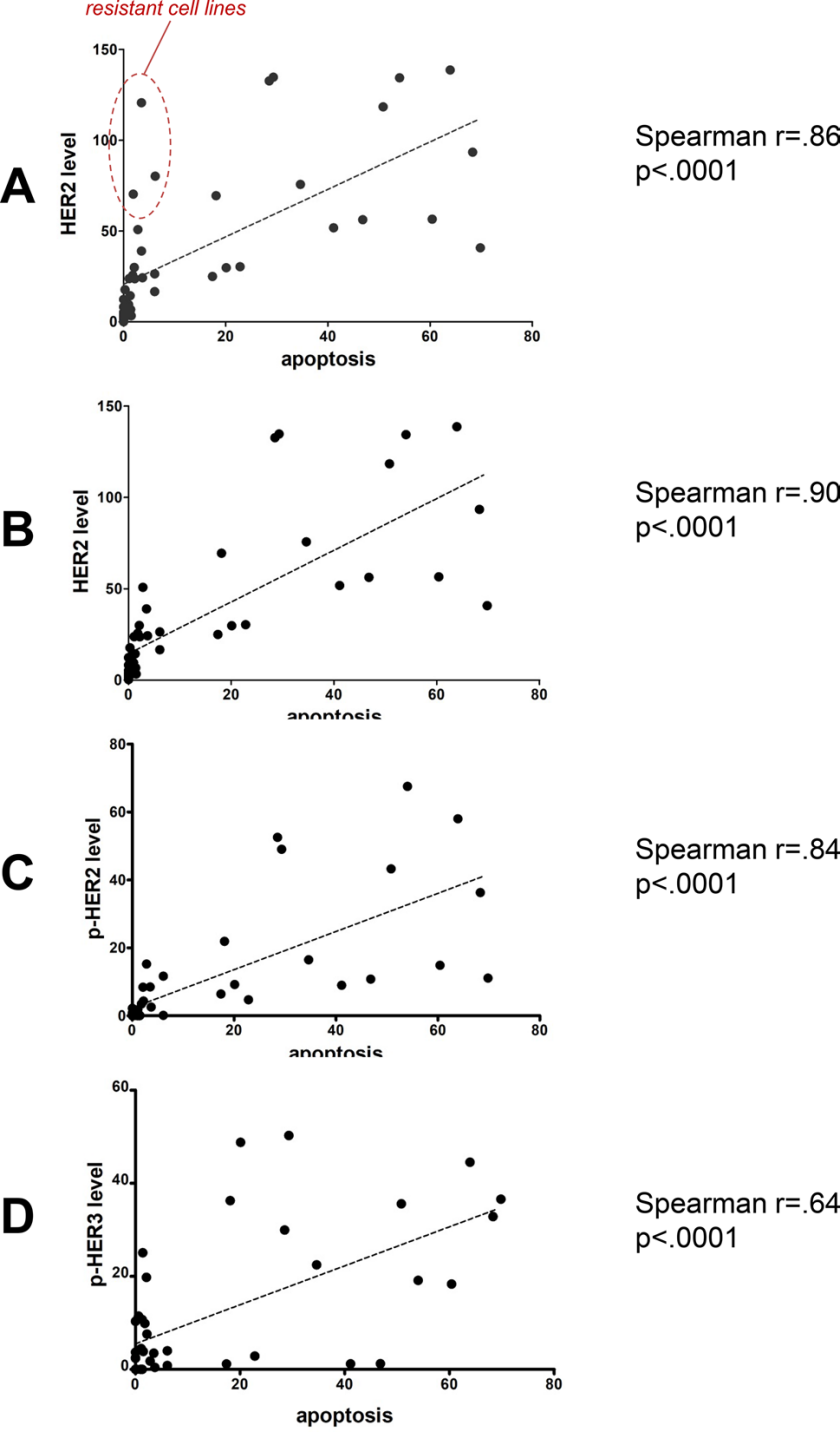
Correlation of HER2 addiction with protein levels. The level of expression of the indicated proteins was quantified by densitometry and plotted against the depth of HER2 addiction (measured as % lapatinib-induced apoptosis). The Spearman correlation coefficients were calculated and are shown for each protein or phosphoprotein. The data points for the 3 cells lines HCC1569, CLS-439, and USPC-ARK1 are shown in the circle. For parts B-D these three were omitted in the analysis.

Most HER2-amplified and HER2 overexpressing breast cancer cells undergo apoptotic cell death following treatment with the HER family selective inhibitor lapatinib (Fig. 3). This correlates with the degree of HER2 protein overexpression. MDA-361 and UACC732, although frequently characterized as HER2 over-expressing cancer cells, have relatively lower expression of HER2 and show little apoptotic effects following lapatinib treatment (Fig. 3). Among all HER2-amplified cancer cells, the apoptotic response to lapatinib generally correlates with the level of HER2 protein overexpression. Cancer cells with high HER2 overexpression such as N87, OE19, TE-4, and USPC-ARK2 show substantial apoptotic response to lapatinib treatment, while others with low levels of HER2 overexpression such as MKN7, OE-33, KYSE-410, and ARK4 do not. The relationship between the level of HER2 expression and the depth of HER2 addiction measured by the amount of lapatinib-induced apoptosis is significant with a Spearman correlation coefficient of 0.86 (Fig. 4A). There are a few cancer cells that have a mechanism-based resistance to lapatinib-induced apoptosis. HCC1569 cells have high HER2 over-expression, but the loss of PTEN in these cells accounts for high constitutive Akt signaling that mediates resistance to lapatinib-induced apoptosis. This has been well described and can be reversed by inhibition of PI3Kα^35^. Two other cell lines that appear to be entirely resistant are CLS-439 bladder cancer and USPC-ARK1 endometrial cancer cells which have high HER2 overexpression but little lapatinib-induced apoptosis. The mechanistic basis for lapatinib resistance in these cells is currently unknown. If these three resistant cells are excluded from the analysis (HCC1569, CLS-439, USPC-ARK1), the relationship between HER2 levels and depth of HER2 addiction is even stronger with a Spearman correlation coefficient of 0.90 (Fig. 4B). None of the negative control cell lines in this panel, which lack HER2 protein overexpression, show an apoptotic response to lapatinib. Similarly, the gastric cell lines with constitutive HER3 phosphorylation (KatoIII, SNU-6, SNU-16, TE-7) do not exhibit an apoptotic response to lapatinib treatment. P-HER2 generally correlates with HER2 levels and similarly correlates with depth of HER2 addiction (Fig. 4C). The level of HER3 phosphorylation also somewhat parallels the levels of HER2 and p-HER2 and similarly shows a reasonable correlation with the depth of HER2 addiction (Fig. 4D). However there is no correlation between level of HER3 expression and the depth of addiction to HER2.

**Figure 5:**
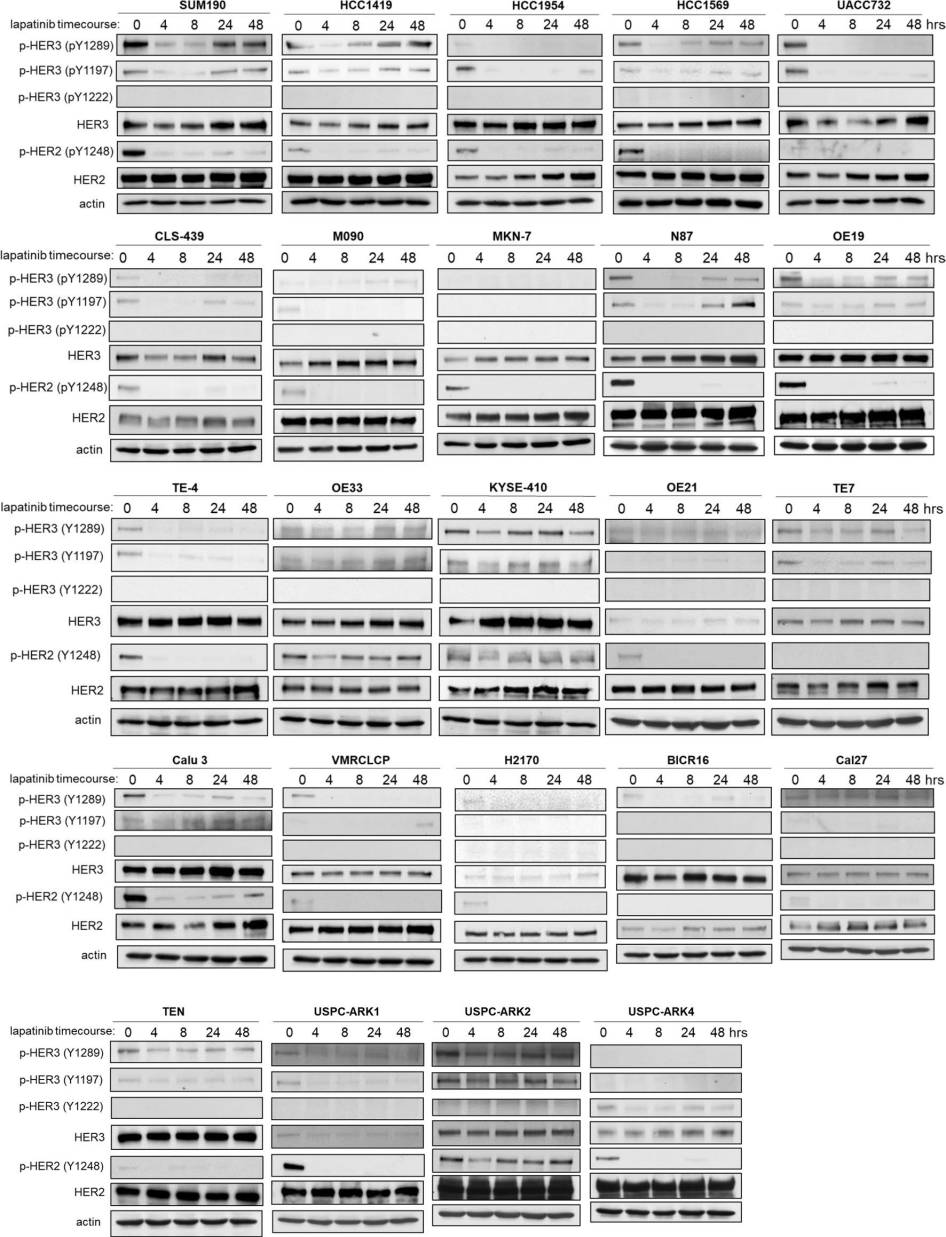
The compensatory dynamics of HER3 signaling in HER2-amplified cancer cells. The indicated cell lines were treated with 200 nM lapatinib for the indicated time course and cell signaling studied by western blotting as shown. The majority of these blots reflect single experiments.

We assessed whether inhibition of HER2 induces compensatory HER3 upregulation or rephosphorylation in these HER2-amplified cancer cells. This was assayed over a 48 h timepoint following lapatinib treatment. Almost all HER2-amplified cancer cells show only a transient HER3 inactivation and HER3 is eventually rephosphorylated over time (Fig. 5). The exception is MKN7 which appears to lack detectable constitutive phosphorylation of HER3. The dynamics of HER3 are quite variable across different cell lines. Some HER2-amplified cell lines (HCC1569, UACC732, CLS439, M090, MKN7, TE4, VMRCLCP, and BICR16) showed complete dephosphorylation of HER3, whereas some cell lines (SUM190, KYSE410, TE7, OE33, Calu3, USPC-ARC2, and TEN) showed partial HER3 dephosphorylation in the short timepoint following lapatinib treatment. However all HER2-amplified cancer cells do show a compensatory increase in HER3 expression, albeit to different extents and with different dynamics. Some cancer cells also show an increase in HER2 protein expression over time (HCC1954, UACC732, MKN7, and BICR16) following lapatinib treatment.

**Figure 6:**
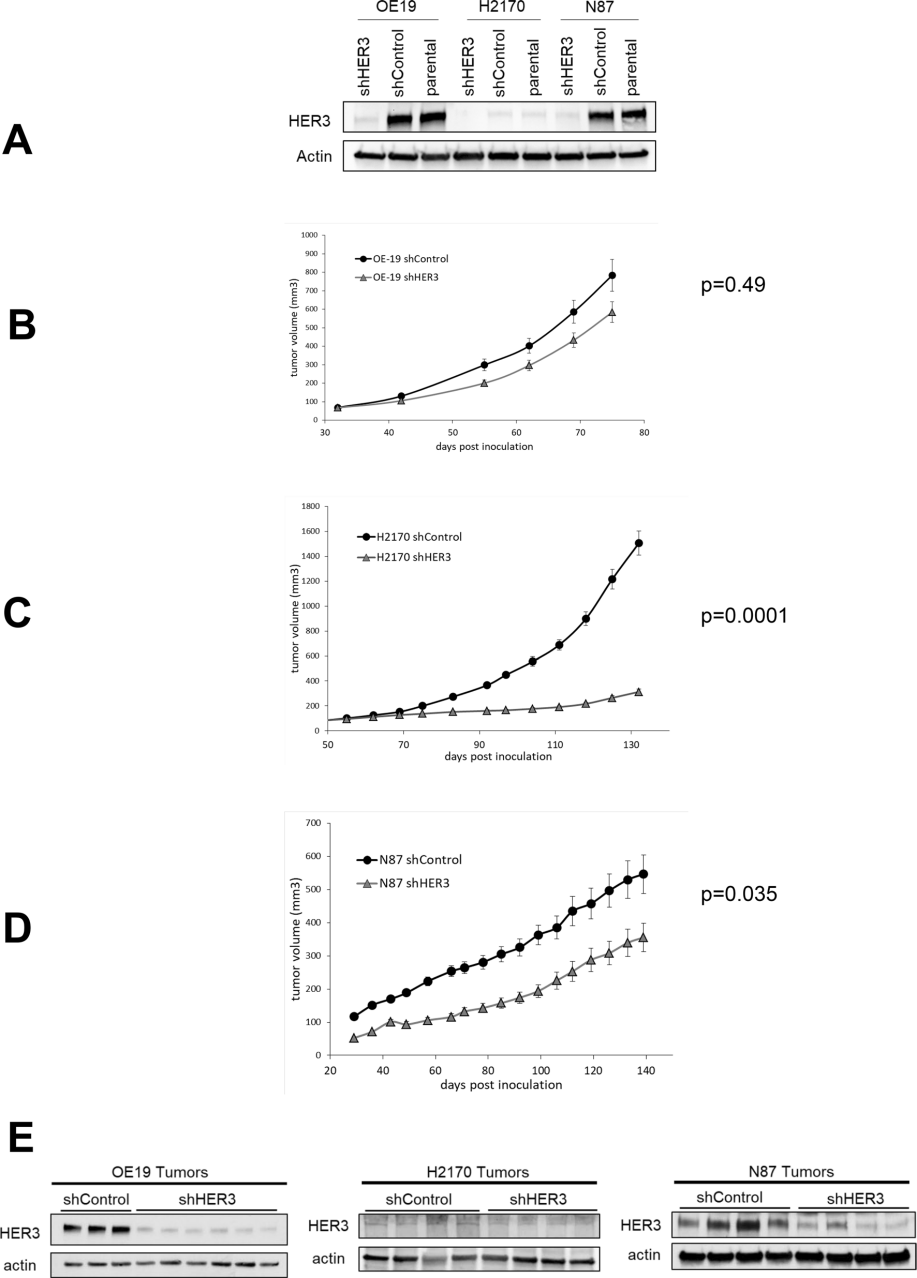
The requirement for HER3 in HER2-amplified tumorigenic growth. (**A**) HER3 expression was knocked down by shRNA targeting in the indicated HER2-amplified cancer cell lines. Control cells express a scrambled shRNA sequence. (**B**–**D**) NSG mice were inoculated subcutaneously with these engineered tumor cells and their in vivo growth rates monitored over the indicated time span. The total sample sizes were n = 19 (OE-19), n = 19 (H2170), and n = 20 (N87) and the errors bars indicate the standard error of the mean. (**E**) Tumors from these mice were harvested at the time of termination of the study and lysates immunoblotted as indicated to confirm the persistent knockdown of HER3 in vivo.

Three HER2-amplified cancer cells from non-breast origin (OE19, N87, and H2170) were selected to determine whether HER3 is essential for tumor growth. H2170 cells were chosen due to their barely detectable HER3 expression in cell culture since in vivo growth may yet involve the conditional or transient expression of HER3. These cells were engineered to express HER3 shRNA leading to effective knock-down of HER3 expression (Fig. 6A). Overall tumor growth was reduced upon HER3 knockdown (Fig. 6B–D). This was most significant in H2170 cells which have the lowest HER3 expression in cell culture. The growth of N87 and OE-19 cancer cells was also suppressed by HER3 knockdown, albeit to a lesser extent. The knockdown was confirmed on the in vivo tumors upon termination of studies (Fig. 6E).

## Discussion

Oncogenes are important biologic drivers of cancer initiation and progression. This has fueled extensive efforts in the field of drug development and cancer therapeutics to treat cancers by targeting their driving oncogenes. This has been successfully translated to practice in the treatment of many cancers including EGFR-driven lung cancers, BRAF-driven melanomas, Bcr-abl driven leukemias, and many other types of cancer. An important lesson learned from the plethora of clinical trials of oncogene-targeting drugs that have been undertaken in the past decades has been that the disease context matters. Targeting an oncogene in one type of cancer may prove highly effective whereas targeting the same oncogene in another type of cancer may be ineffective. Targeting the BRAF V600E oncogene with a single BRAF inhibitor shows response rates of approximately 50% in melanomas or non-small cell lung cancers, but no activity in colorectal cancers, anaplastic thyroid cancers, or several other types of cancers^36–38^. PI3K inhibitors have significant efficacy in the treatment of PIK3CA-mutant breast cancers but far less activity in PIK3CA-mutant cancers of other organs^39,40^. Therefore it is apparent that oncogenes function in a context dependent manner and their biological roles are intimately linked with tissue specific signaling.

In this work, we sought to explore the role of the HER2-HER3 tumor driver in disease contexts outside of breast cancers. HER2 amplification was originally described in breast cancers and extensive work has been done to understand HER2 signaling, biology, and therapeutics in this disease context. Particularly revealing has been the role of HER3 as an essential partner for HER2 in breast cancers and extensive efforts are ongoing to develop effective inhibitors of HER3 for combination HER2 and HER3 targeting of this disease. Whether these concepts and applications are relevant to HER2-amplified cancers originating in other organs remains unknown and our study is an effort in a limited set of cancer cell lines to begin to explore this knowledge gap. Our study confirms the biological relevance of amplified and overexpressed HER2 across diverse tumor types, but some differences are suggested. We found that the level of HER2 protein overexpression is linked with the degree of tumor addiction to HER2 such that tumors that are considered HER2 overexpressing, but at lower levels of expression and autophosphorylation than seen in other tumors, appear to have less apoptotic response to lapatinib. This implies that the concept of a driver oncogene is not a binary designation, but rather can manifest along a gradient of oncogene dependencies. This is unique to oncogenes activated through amplification and overexpression, such as HER2, and lies in sharp contrast to oncogenes activated through mutational or fusion events. We observed that the expression and role of HER3 is not the same across the spectrum of HER2-amplified cancers. Although ubiquitously expressed in HER2-amplified breast cancers, its expression is variable in other cancers and undetectable in some cancer cells. However the level of HER3 protein expression does not reflect the dependency on HER3. This is best seen with H2170 lung cancer cells in which HER3 expression is barely detectable in tissue culture, but their tumorigenic growth is clearly stunted by expression of HER3 shRNA. On the other hand N87 and OE-19 gastric cancer cells have high expression of HER3 but their tumorigenic growth is less affected by HER3 knockdown. In breast cancers, the HER3 dependency is well established and shRNA knockdowns and Crispr knockouts of HER3 have shown complete suppression of in vivo growth in multiple HER2-amplified breast cancer cell lines^9,31,41^.

The expression of HER3 is dynamic and significantly induced in HER2-amplified breast cancers when treated with HER2 inhibitors^13–15^. In this study, we find that the compensatory increase in HER3 expression or phosphorylation and/or the restoration of HER3 phosphorylation with prolonged lapatinib treatment is seen in some, but not all HER2-overexpressing cancers. The different extent and dynamics of this compensatory response is only consistent with the multitude of mechanisms that mediate it, and which may vary dependent on the cell context^11^. This highlights the differences in circuitry between cancer types and between individual cases of cancer. There is also a lapatinib induced increase in total HER2 levels in some, but not other, cancer cell lines. This has been reported with lapatinib treatment in some cell lines^42^.

Collectively these data do not support a uniform role for HER3 in all types of HER2-amplified cancers and suggest that its role is dependent on the disease context, and possibly even on the specific case context. These data also reveal the lack of direct link between the expression of HER3 and its functional importance suggesting that the expression of HER3 may not be a valid biomarker for emerging HER3 inhibitors. There are a number of HER3 inhibitors in the pharmaceutical pipelines including antibodies that target the extracellular domain of HER3 and small molecules targeting kinase domain or other functions of HER3^43–53^. The evidence suggests that HER3 is a valid co-target in the treatment of all HER2-amplified breast cancers. But this work suggests that HER3 does not have the same essential function in all HER2-amplified cancers of other tissue types, and the expression of HER3 is not a valid biomarker for its functional relevance.

The data do further strengthen the validity of HER2 expression levels as a predictive biomarker. These data support the notion that the depth of addiction to HER2 and thus the sensitivity to HER2 inhibitors correlates well with the level of HER2 overexpression. This will be relevant as more effective HER2 inhibitors are developed that can more potently inactivate this resilient target.

## Methods

### Cell culture and reagents

A total of forty-six cancer cell lines were used in this study, which comprised of 12 breast cancer cell lines (T47D, MDA-231, MCF7, SkBr3, BT474, MDA-361, MDA-453, HCC1569, HCC1954, HCC1419, UACC732, and SUM190), 2 bladder cancer cell lines (T24 and CLS-439), 3 colon cancer cell lines (HCT-116, HT29, and M090), 8 gastric cancer cell lines (AGS, KatoIII, SNU-1, SNU-5, SNU-16, MKN-7, N87, and OE19), 7 esophageal cancer cell lines (TE-7, OE-21, BIC-1, SEG-1, TE-4, OE-33, and KYSE-410), 6 lung cancer cell lines (H460, H1703, H838, VMRCLCP, H2170, and Calu3), 2 tongue cancer cell lines (Cal27 and BICR-16), and 6 endometrium cancer cell lines (TEN, USPC-ARC1, USPC-ARC2, USPC-ARC4, USPC-ARC7, and Hec1A). The source of each cell line is described in Supplementary table 1. All cells tested negative for mycoplasma contamination by polymerase chain reaction (PCR) and were subsequently grown in fresh medium (RPMI1640, DMEM/F12, Myco's 5A, MEM) supplemented with 10% fetal bovine serum (Gemini Bio-Products) and penicillin and streptomycin (ThermoFisherScientific). Lapatinib was purified from Tykerb tablets by organic extraction as previously described^13^.

### Apoptosis assays

Cells were washed three times in PBS and stained with an apoptosis detection kit (BMS500FI/300, eBioscience) consisting of FITC-conjugated annexin V and propidium iodide (PI) following the vendor’s recommendation. The stained samples were assayed using an LSR II (BD) flow cytometer and analyzed using FlowJo software.

### Generation of HER3 knockdown cells

Small hairpin RNA (shRNA) sequences (HER3-specific and scrambled) were cloned into pSuperior vector (Oligoengine) containing a neomycin resistance cassette as a selectable marker. Then both shRNAs (HER3-specific and scrambled) were transfected into Phoenix cells using Lipofectamine 2000 reagent as per recommended protocol provided with the kit. After 72Hrs of transfection, media containing live virus was collected and filtered using a 0.45uM filter. Afterward, this viral supernatant was concentrated using Retro-X concentrator as per manufacturer protocol. Next, the viral particle was diluted in the respective media, combined with polybrene (4ug/ml), and then added to the cells (OE19, N87, and H2170) that have been seeded the previous day. After infection, cells were allowed to recover for 72hrs and selected in G418 (400 μg/ml) antibiotic. The shRNA sequences are below.

HER3 shRNA:

5′-AGCTTAAAAAAAGAGGATGTCAACGGTTATCTCTTGAATAACCGTTGACATCCTCTTGG-3’.

control shRNA:

5′-AGCTTTTCCAAAAACCTAAGGTTAAGTCGCCCTTCTCTTGAAAGGGCGACTTAACCTTAGGGG-3’.

### Western blotting and antibodies

Western blot analysis was performed as previously described^31^. Briefly, cell lysates were made in mRIPA buffer (containing 150 mmol/L NaCl, 0.1% SDS, 1% Nonidet P40, 1% sodium deoxycholate and 10 mmol/L sodium phosphate, pH 7.2), which was supplemented with leupeptin, aprotinin, phenylmethylsulfonyl fluoride (PMSF), sodium vanadate, and phosphatase inhibitor cocktail (Roche # 04906845001). The protein concentrations were estimated by the Pierce BCA assay (ThermoFisherScientific). Equal amounts of protein (50 ugs) were resolved on 8–10% denaturing SDS polyacrylamide gel and transferred onto a PVDF membrane. The membranes were blocked with 3% bovine serum albumin (BSA), stained with primary antibodies overnight at 4 °C, washed 3 times with TBST (Tris-buffered saline containing 1% Triton-X-100), stained with secondary antibodies for 1 h at room temperature and detected using chemiluminescence. Antibodies for HER3 (5A12) (#81,455), HER2 (C18) (#284), actin (#1616) were purchased from Santa Cruz Biotechnology. Antibodies for p-HER2Y1248 (#2247), p-HER3Y1289 (#4791S), p-HER3Y1197 (#4561S), p-HER3Y1222 (#4784S), pAKT-T308 (#4056)/ pAKT-S473 (#4058), p-MAPK (#9101), MAPK (#9102) were purchased from Cell Signaling Technology. Horseradish peroxidase-conjugated secondary antibodies were from Santa Cruz Biotechnology (mouse and goat) and GE Healthcare (rabbit).

### HER2 gene amplification assay

HER2 gene amplification was quantified using Gastrin as a chromosome 17 reference gene as has been previously reported and validated^32–34^. Genomic DNA was purified from cells using Qiagen kits. 10 ng of genomic DNA was used in a real-time multiplex PCR reaction using 500 μm of HER2 primers (Fwd: ATCTGCCTGACATCCACG; Rev: GCAATCTGCATACACCAGTTC), 700 μM of Gastrin primers (Fwd: TCTGAAGCTTCTTGGAAGCC; Rev: CCAGCTGCCTTCGATGA), and 250 μM of Taqman probes for HER2 (6FAM- AGCTTATGCCCTATGGCT-MGBNFQ) and Gastrin (VIC-AGATGCACCCTTAGGTACA-MGBNFQ) in the Taqman master mix (Thermofisher). Reactions were run on a BioRad iCycler realtime detection system for one cycle at 95C × 10 min followed by 60 cycles of 95C × 15 s, 60C × 30 s, 72C × 10 s and calculated using the Biorad C1000 manager software. Data are shown as a ratio of HER2/Gastrin genes.

### Mouse xenograft growth assays

All mice studies were reviewed and approved by the UCSF Institutional Animal Care and Use Committee (IACUC) and conducted in accordance with the relevant guidelines and regulations. These experiments are reported in accordance with the ARRIVE Guidelines. A total of 2 × 10^6^ OE19, N87, H2170 cells (100 μl, 1:1 matrigel: serµm-free RPMI) were implanted subcutaneously in the flanks of female NSG mice (NOD SCID gamma mouse) at 6 to 8 weeks of age. Approximately 20 age-matched mice were used for each tµmor model and assigned randomly to each experimental group using the stratified randomization model of the StudyLog Desktop software. Tµmor growth was assessed weekly by caliper measurement.

From each group mice (HER3 shRNA and Control shRNA), 4 tumor samples were collected and stored at − 80 °C until further use. On the final day, all samples were removed from the freezer and placed in dry ice. Then samples were pulverized using BioPulverizer and liquid nitrogen to make powder. Later, samples were mixed with mRIPA buffer and kept 30 mins on ice. After 30 mins, the samples were spun down at 14,000 rpm for 10 min (4 °C) and the clear supernatants were quantified using the BCA methods and analyzed by SDS-PAGE and immunoblotting.

### Statistical analysis

For comparison of the arms in the mouse xenograft assays, the area under the curve for each mouse was computed after taking the square root of volume measurements and the difference in AUCs between the two groups was calculated using the Wilcoxon rank sum test. For the HER2/p-HER2/HER3 correlations with apoptosis, the Spearman coefficient values were calculated and the graph was plotted using GraphPad Prism.

## Supplementary Information

Supplementary Information
