# Supplementary material for: The role of HER2 and HER3 in HER2-amplified cancers beyond breast cancers
Source: Sci Rep. 2021 Apr 27;11:9091. doi: 10.1038/s41598-021-88683-w (PMC8079373; doi:10.1038/s41598-021-88683-w)

Title

The role of HER2 and HER3 in HER2-amplified cancers beyond breast cancers

Authors

Avisek Majumder[1], Manbir Sandhu[2], Debarko Banerji[3], Veronica Steri[4], Adam Olshen[4,5], Mark M. Moasser[1,4]

Supplementary Materials

Supplementary Table 1

Supplementary Full gel figures

Supplementary Table 1

Sources of the cell lines

	Source	Location	comments
T47D	ATCC	Manassas, Virginia	
MDA-231	ATCC	Manassas, Virginia	
MCF-7	ATCC	Manassas, Virginia	authenticated June 2019; Genetica Cell Line te
SkBr3	ATCC	Manassas, Virginia	authenticated June 2019; Genetica Cell Line te
BT474	ATCC	Manassas, Virginia	authenticated June 2019; Genetica Cell Line te
MDA-361	ATCC	Manassas, Virginia	
MDA-453	ATCC	Manassas, Virginia	
HCC1569	ATCC	Manassas, Virginia	authenticated June 2019; Genetica Cell Line te
HCC1954	ATCC	Manassas, Virginia	
HCC1419	ATCC	Manassas, Virginia	
UACC732	ATCC	Manassas, Virginia	
SUM190	Asterand	Detroit, MI	
T24	ATCC	Manassas, Virginia	
CLS-439	Cell Line Service	Eppelheim, Germany	within 6 months of purchase
HCT116	gift of Michael Korn	UCSF	
HT29	ATCC	Manassas, Virginia	within 6 months of purchase
M090	gift of Livio Trusolini	Univ of Turin	(converted from PDX, not a published cell line
AGS	ATCC	Manassas, Virginia	within 6 months of purchase
KatoIII	ATCC	Manassas, Virginia	within 6 months of purchase
SNU-1	ATCC	Manassas, Virginia	within 6 months of purchase
SNU-5	ATCC	Manassas, Virginia	within 6 months of purchase
SNU-16	ATCC	Manassas, Virginia	within 6 months of purchase
MKN7	JCRB Cell Bank	Osaka, Japan	within 6 months of purchase
N87	ATCC	Manassas, Virginia	within 6 months of purchase
OE19	gift of Michael Korn	UCSF	
TE-7	gift of Michael Korn	UCSF	
OE-21	gift of Michael Korn	UCSF	
BIC-1	gift of Michael Korn	UCSF	
SEG-1	gift of Michael Korn	UCSF	
TE-4	Riken BRC	Ibaraki, Japan	within 6 months of purchase
OE-33	gift of Michael Korn	UCSF	
KYSE-410	SigmaAldrich	St. Louis, MO	within 6 months of purchase
H460	gift of David Jablons	UCSF	
H1703	gift of David Jablons	UCSF	
H838	gift of David Jablons	UCSF	
VMRCLCP	JCRB Cell Bank	Osaka, Japan	within 6 months of purchase
H2170	ATCC	Manassas, Virginia	within 6 months of purchase
Calu3	gift of Trever Bivona	UCSF	
Cal27	ATCC	Manassas, Virginia	within 6 months of purchase
BICR-16	SigmaAldrich	St. Louis, MO	within 6 months of purchase
TEN	Riken BRC, Ibaraki, Japan		within 6 months of purchase
USPC-ARK1	gift of Allesandro Santin	Yale	
USPC-ARK2	gift of Allesandro Santin	Yale	
USPC-ARK4	gift of Allesandro Santin	Yale	
USPC-ARK7	gift of Allesandro Santin	Yale	
Hec1A	gift of Linda Guidice	UCSF	

ATCC = American Type Culture Collection
JCRB = Japanese Collection of Research Bioresources
Riken BRC = Riken Bioresource Center

Supplementary information

(Full Western blot images for main figures in the paper)

Full-blots for Figure 5

Fig.5: SUM190 cells

P-HER3 (pY1289)

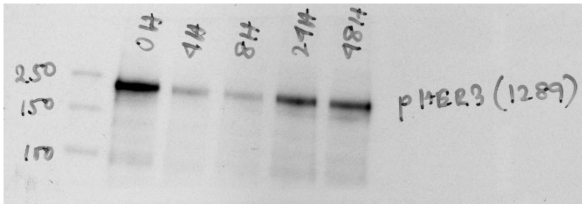

-185

Fig.5: SUM190 cells

P-HER3 (pY1197)

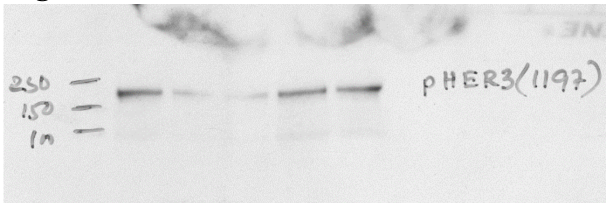

-185

Fig.5: SUM190 cells

P-HER3 (pY1222)

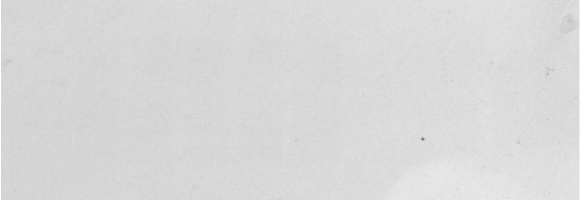

-185

Fig.5: SUM190 cells

HER3

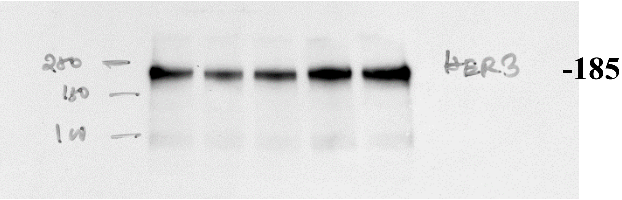

-185

Fig.5: SUM190 cells

P-HER2 (pY1248)

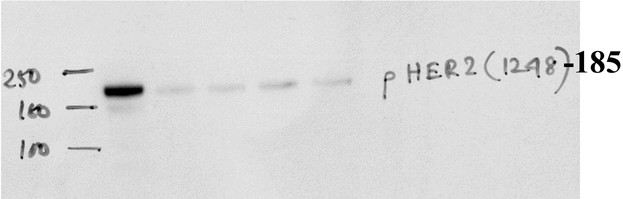

-185

Fig.5: SUM190 cells

HER2

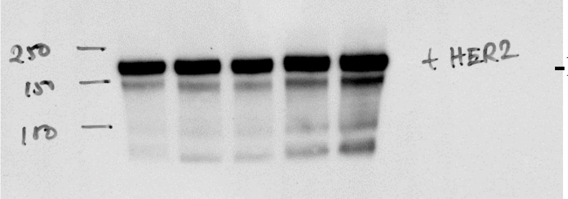

-185

Fig.5: SUM190 cells

Actin

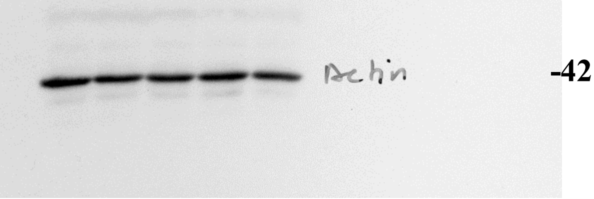

-42

Fig.5: HCC1419 cells

P-HER3 (pY1289)

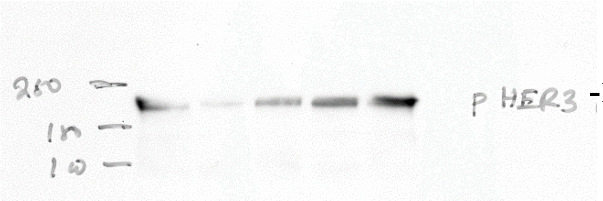

-185

Fig.5: HCC1419 cells

P-HER3 (pY1197)

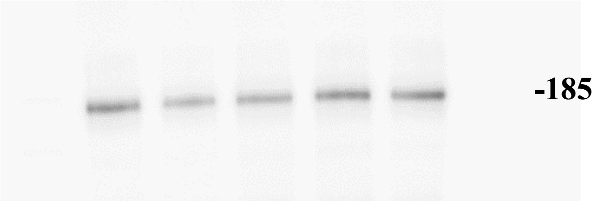

-185

Fig.5: HCC1419 cells

P-HER3 (pY1222)

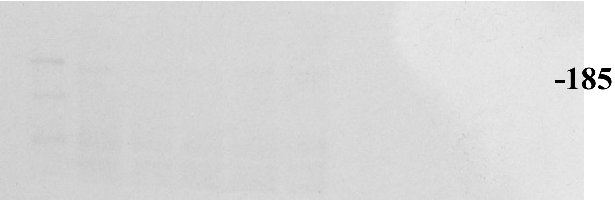

-185

Fig.5: HCC1419 cells

HER3

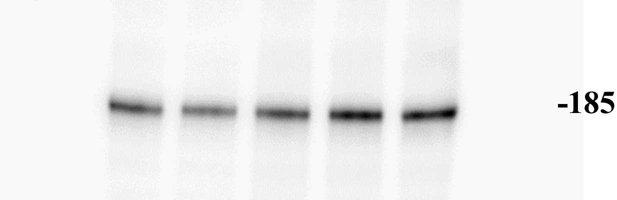

-185

Fig.5: HCC1419 cells

P-HER2 (pY1248)

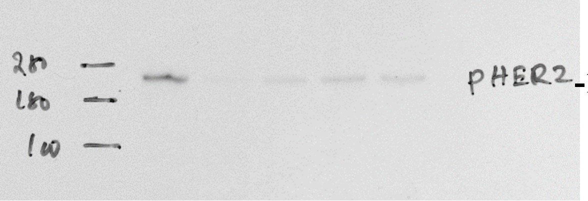

-185

Fig.5: HCC1419 cells

HER2

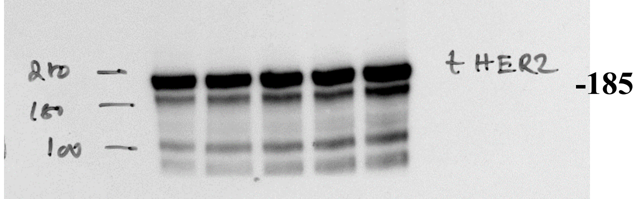

-185

Fig.5: HCC1419 cells

Actin

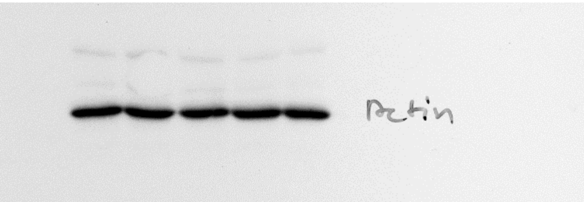

-42

Full-blots for Figure 5

Fig.5: HCC1954 cells

P-HER3 (pY1289)

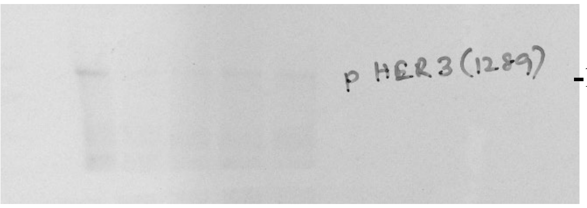

-185

Fig.5: HCC1954 cells

P-HER3 (pY1197)

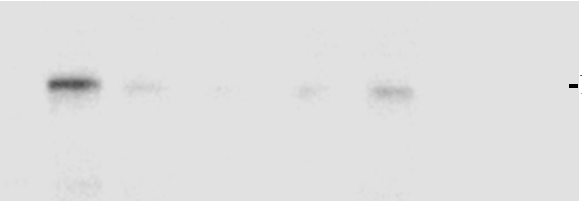

-185

Fig.5: HCC1954 cells

P-HER3 (pY1222)

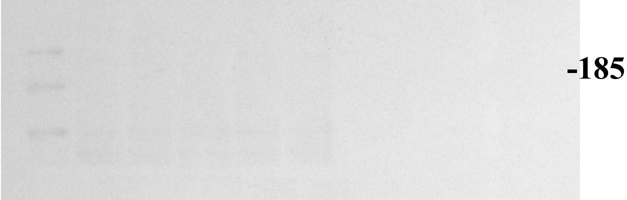

-185

Fig.5: HCC1954 cells

HER3

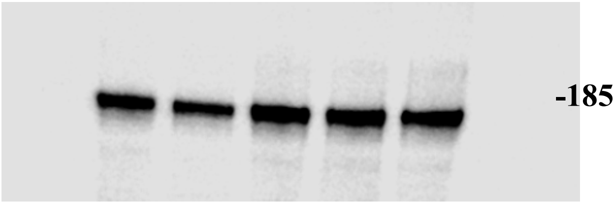

-185

Fig.5: HCC1954 cells

P-HER2 (pY1248)

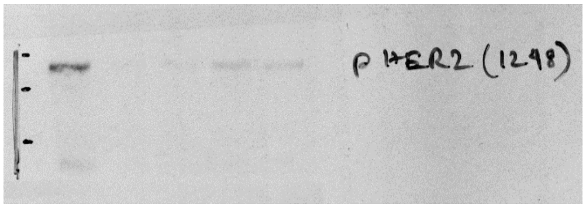

-185

Fig.5: HCC1954 cells

HER2

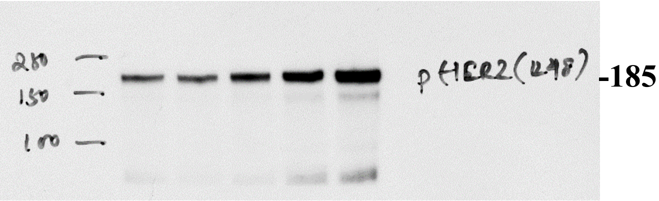

-185

Fig.5: HCC1954 cells

Actin

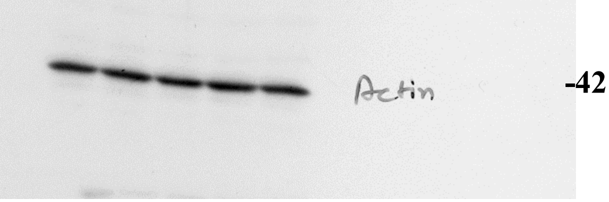

-42

Fig.5: HCC1569 cells

P-HER3 (pY1289)

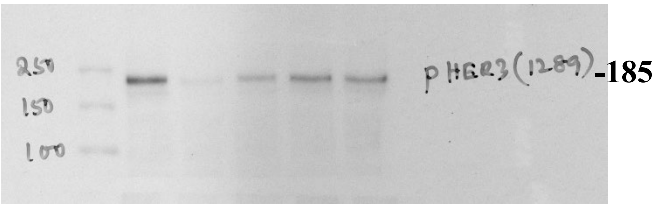

-185

Fig.5: HCC1569 cells

P-HER3 (pY1197)

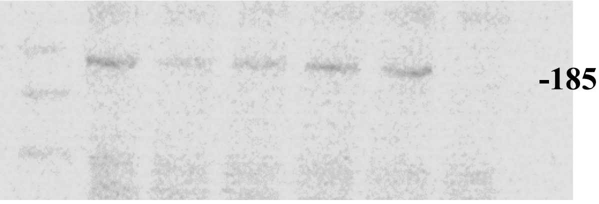

-185

Fig.5: HCC1569 cells

P-HER3 (pY1222)

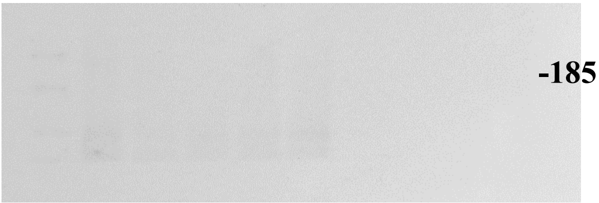

-185

Fig.5: HCC1569 cells

HER3

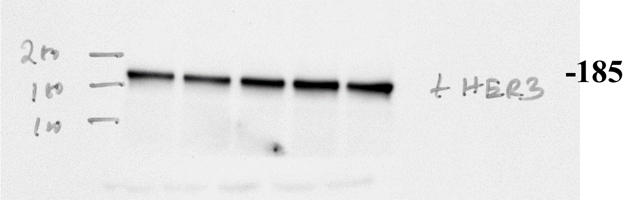

-185

Fig.5: HCC1569 cells

P-HER2 (pY1248)

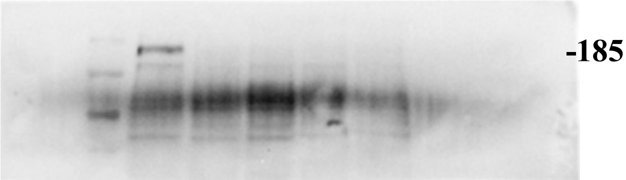

-185

Fig.5: HCC1569 cells

HER2

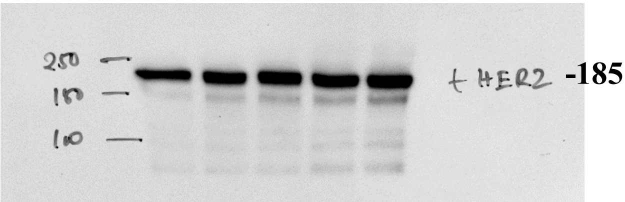

-185

Fig.5: HCC1569 cells

Actin

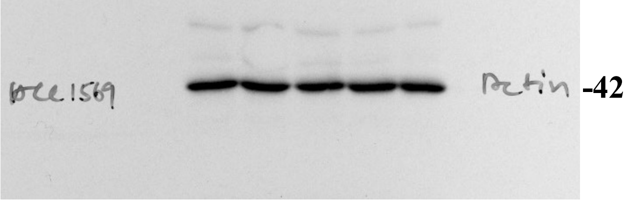

-42

Full-blots for Figure 5

Fig.5: UACC732 cells

P-HER3 (pY1289)

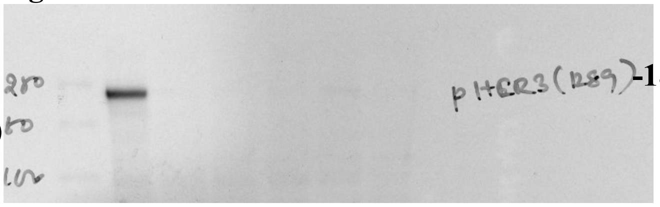

-185

Fig.5: UACC732 cells

P-HER3 (pY1197)

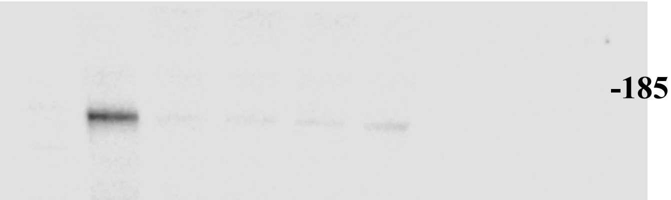

-185

Fig.5: UACC732 cells

P-HER3 (pY1222)

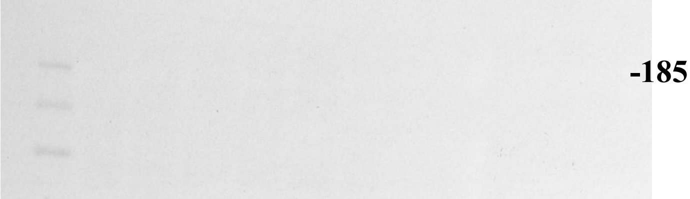

-185

Fig.5: UACC732 cells

HER3

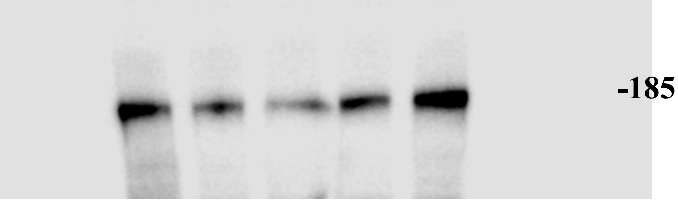

-185

Fig.5: UACC732 cells

P-HER2 (pY1248)

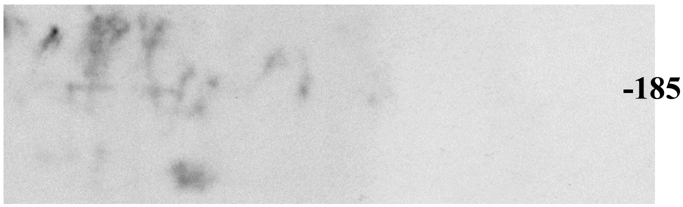

-185

Fig.5: UACC732 cells

HER2

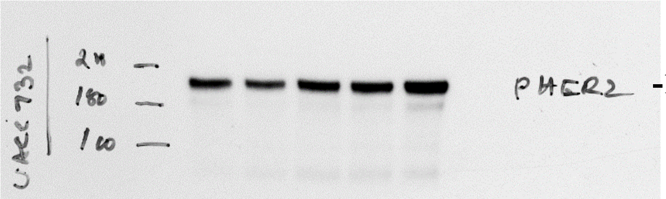

-185

Fig.5: UACC732 cells

Actin

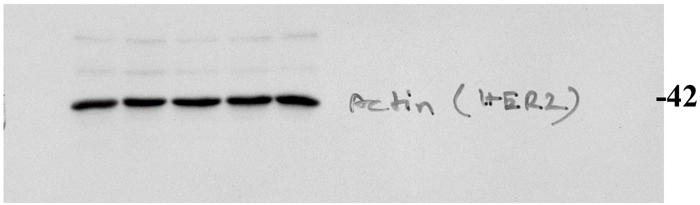

-42

Fig.5: CLS-439 cells

P-HER3 (pY1289)

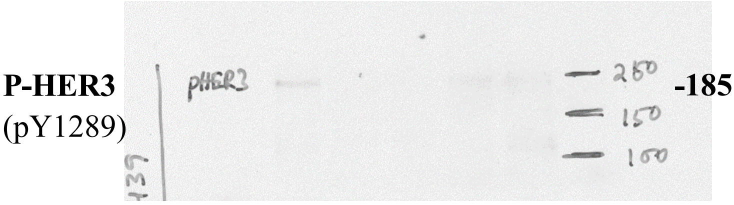

-185

Fig.5: CLS-439 cells

P-HER3 (pY1197)

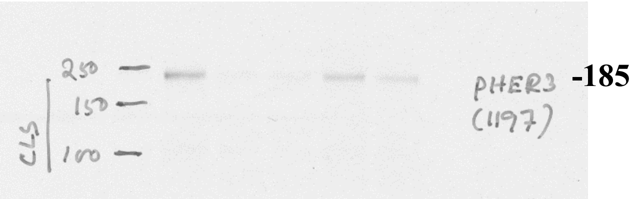

-185

Fig.5: CLS-439 cells

P-HER3 (pY1222)

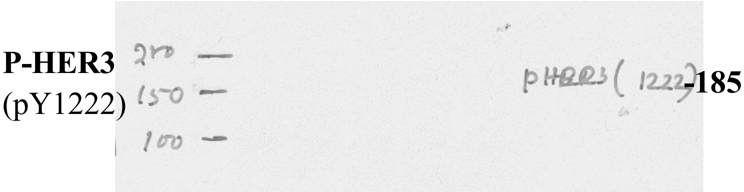

-185

Fig.5: CLS-439 cells

HER3

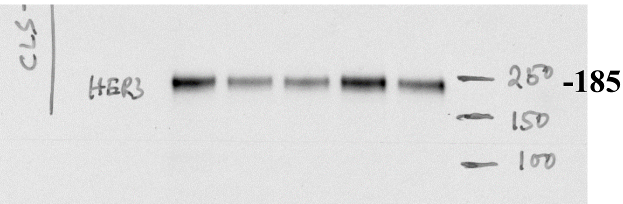

-185

Fig.5: CLS-439 cells

P-HER2 **(pY1248)**

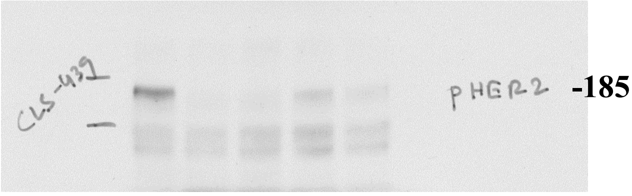

-185

Fig.5: CLS-439 cells

HER2

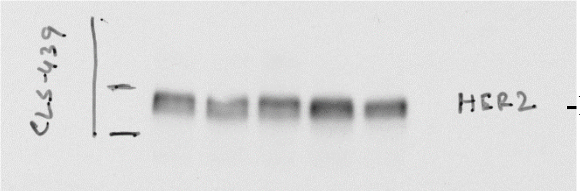

-185

Fig.5: CLS-439 cells

Actin

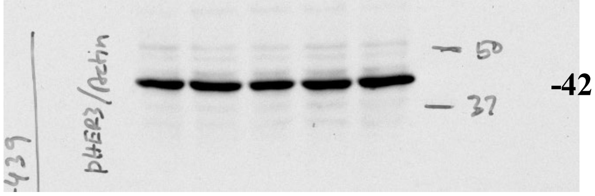

-42

Full-blots for Figure 5

Fig.5: M090 cells

P-HER3 (pY1289)

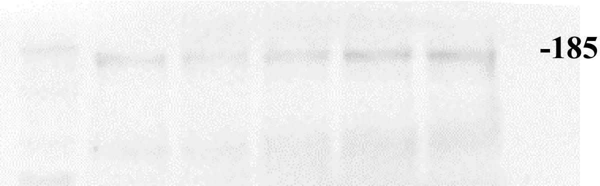

-185

Fig.5: M090 cells

P-HER3 (pY1197)

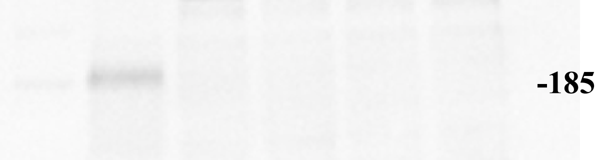

-185

Fig.5: M090 cells

P-HER3 (pY1222)

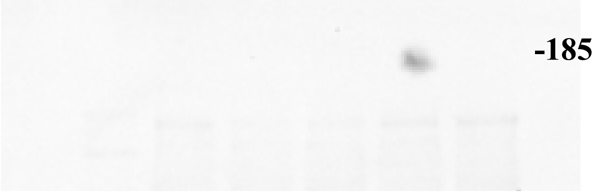

-185

Fig.5: M090 cells

HER3

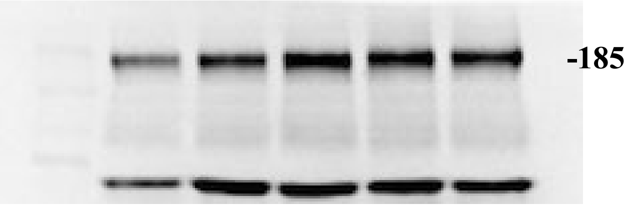

-185

Fig.5: M090 cells

P-HER2 (pY1248)

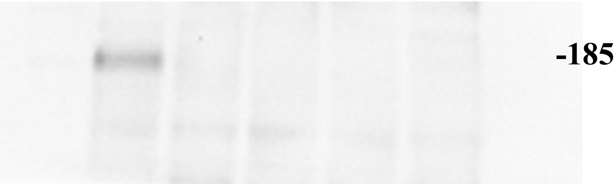

-185

Fig.5: M090 cells

HER2

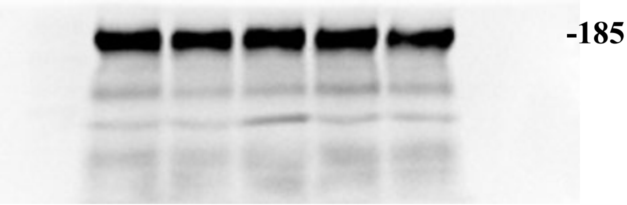

-185

Fig.5: M090 cells

Actin

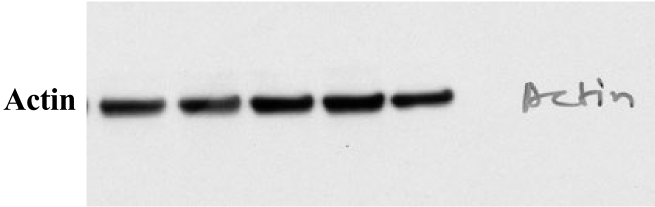

-42

Fig.5: MKN-7 cells

P-HER3 (pY1289)

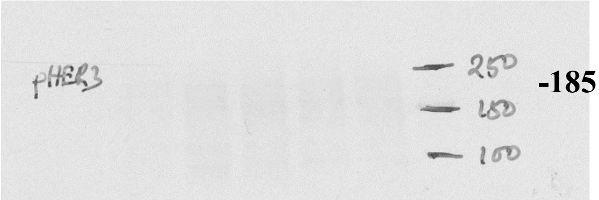

-185

Fig.5: MKN-7 cells

P-HER3 (pY1197)

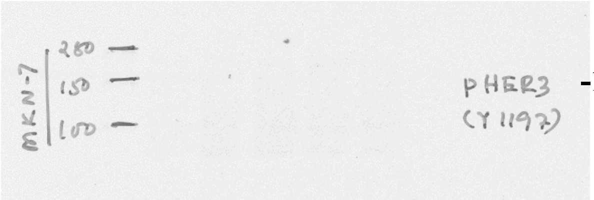

-185

Fig.5: MKN-7 cells

P-HER3 (pY1222)

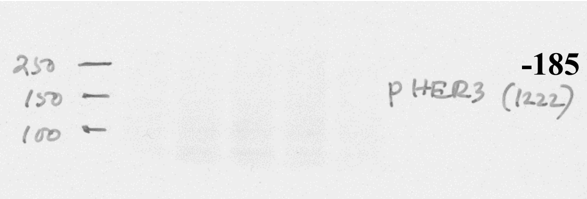

-185

Fig.5: MKN-7 cells

HER3

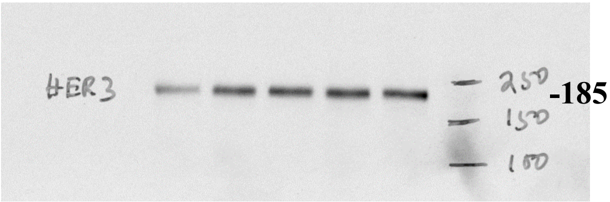

-185

Fig.5: MKN-7 cells

P-HER2 (pY1248)

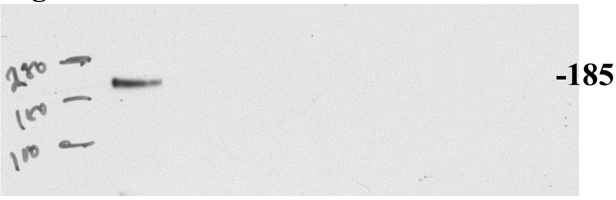

-185

Fig.5: MKN-7 cells

HER2

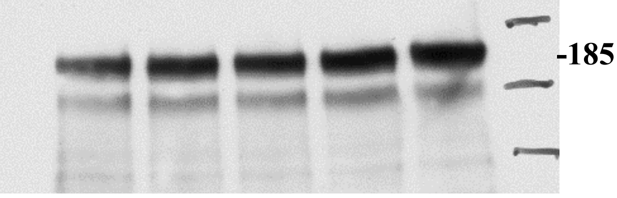

-185

Fig.5: MKN-7 cells

Actin

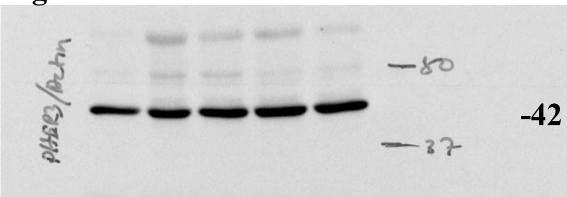

-42

Full-blots for Figure 5

Fig.5: N87 cells

P-HER3 (pY1289)

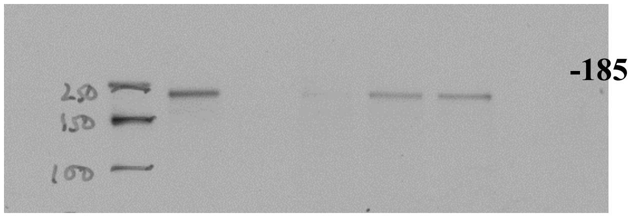

-185

Fig.5: N87 cells

P-HER3 (pY1197)

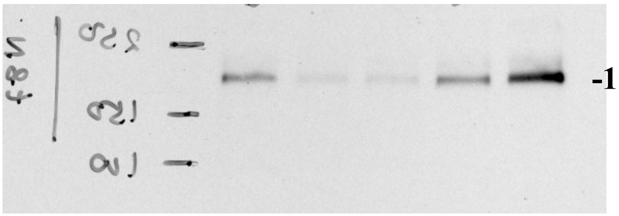

-185

Fig.5: N87 cells

P-HER3 (pY1222)

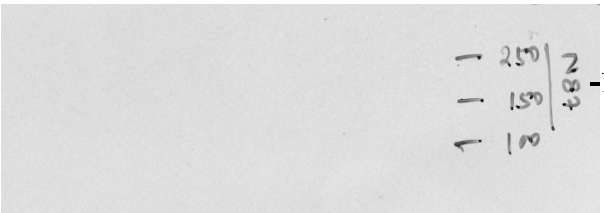

-185

Fig.5: N87 cells

HER3

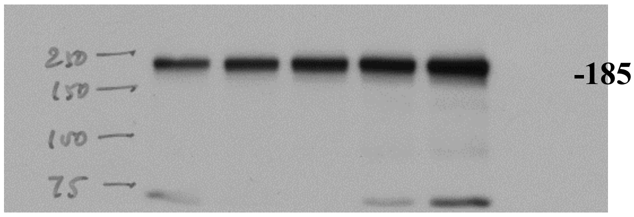

-185

Fig.5: N87 cells

P-HER2 (pY1248)

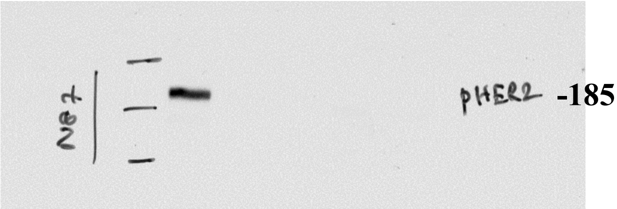

-185

Fig.5: N87 cells

HER2

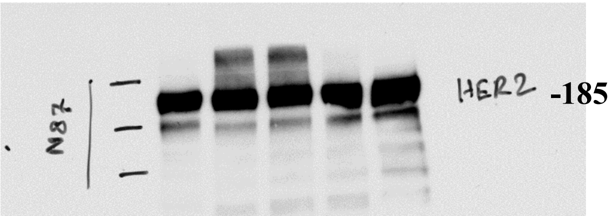

-185

Actin

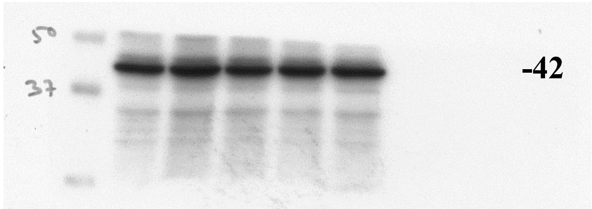

-42

Fig.5: OE19 cells

P-HER3 (pY1289)

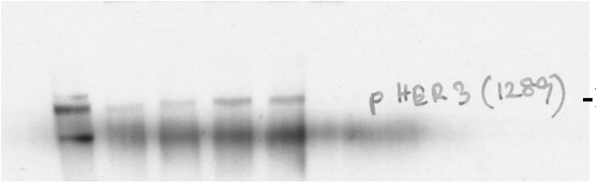

-185

Fig.5: OE19 cells

P-HER3 (pY1297)

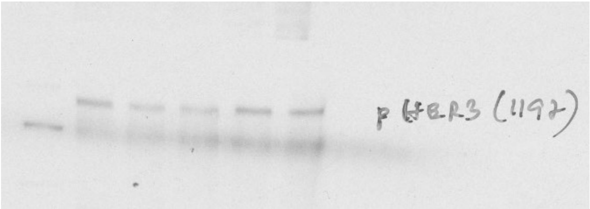

-185

Fig.5: OE19 cells

P-HER3 (pY1222)

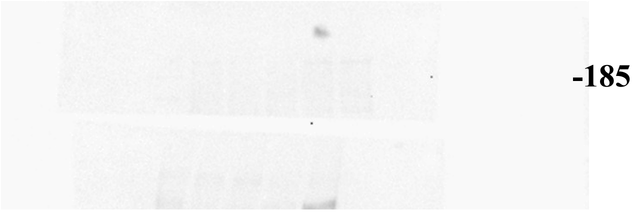

-185

Fig.5: OE19 cells

HER3

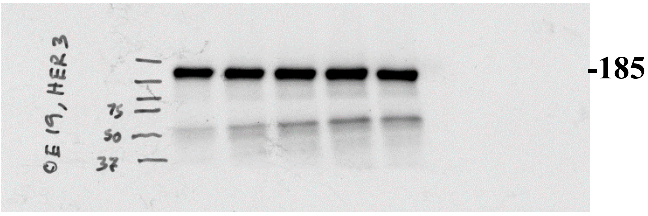

-185

Fig.5: OE19 cells

P-HER2 (pY1248)

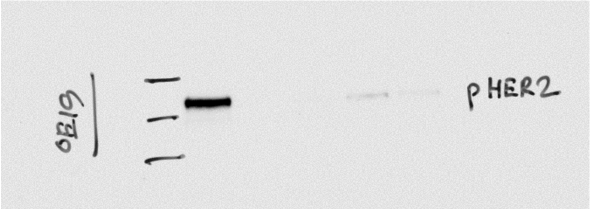

-185

Fig.5: OE19 cells

HER2

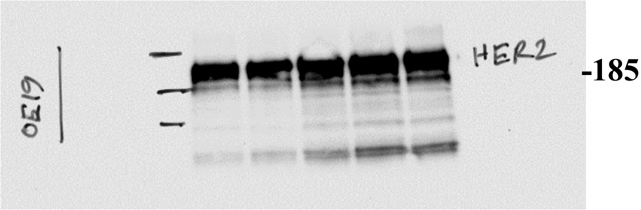

-185

Actin

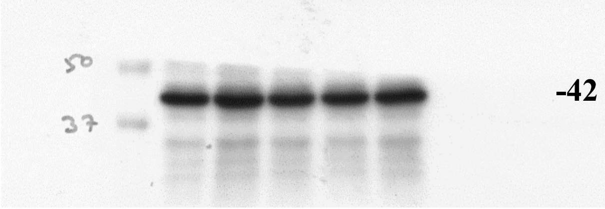

-42

Full-blots for Figure 5

Fig.5: TE-4 cells

P-HER3 (pY1289)

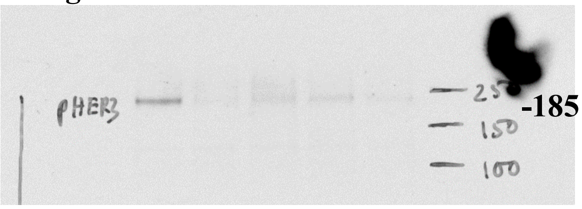

-185

Fig.5: TE-4 cells

P-HER3 (pY1197)

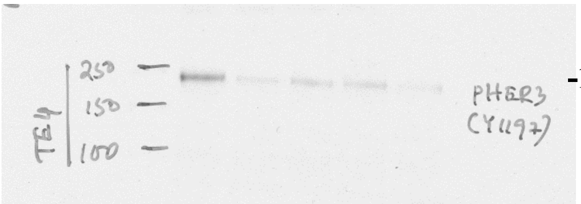

-185

Fig.5: TE-4 cells

P-HER3 (pY1222)

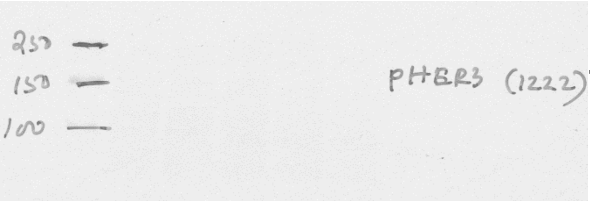

-185

Fig.5: TE-4 cells

HER3

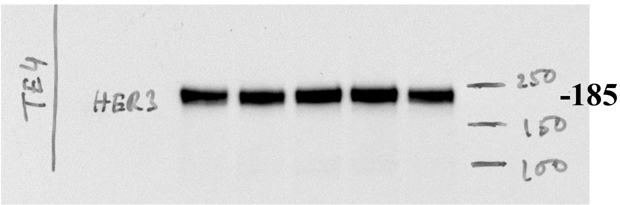

-185

Fig.5: TE-4 cells

P-HER2 (pY1248)

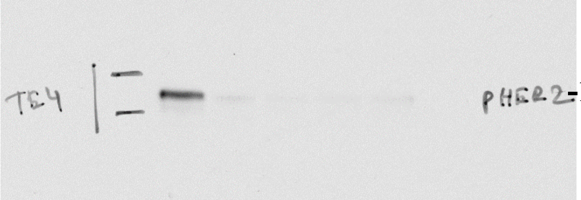

-185

Fig.5: TE-4 cells

HER2

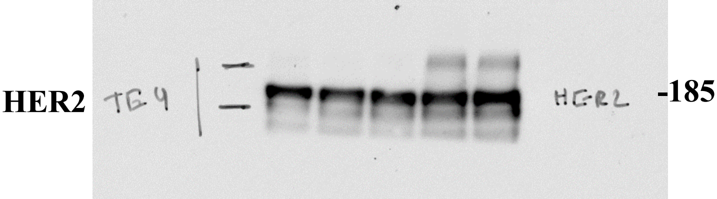

-185

Fig.5: TE-4 cells

Actin

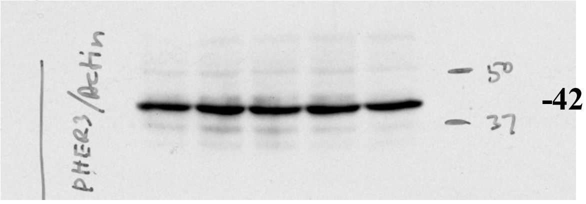

-42

Fig.5: OE33 cells

P-HER3 (pY1289)

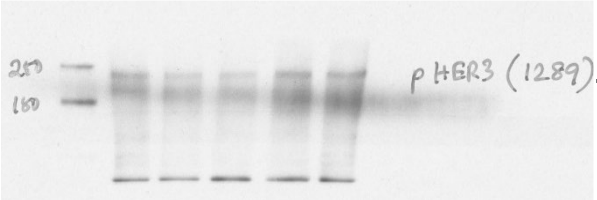

-185

Fig.5: OE33 cells

P-HER3 (pY1197)

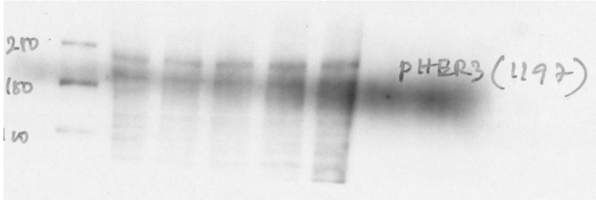

-185

P-HER3 (pY1222)

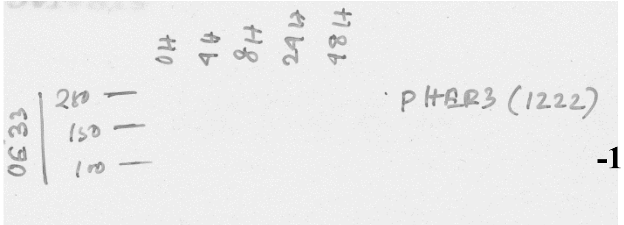

-185

Fig.5: OE33 cells

HER3

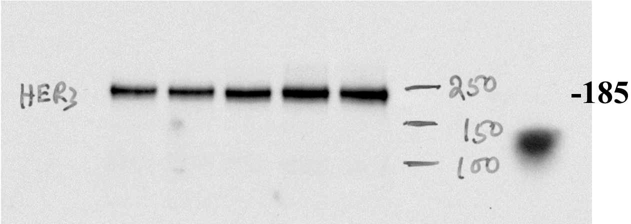

-185

Fig.5: OE33 cells

P-HER2 (pY1248)

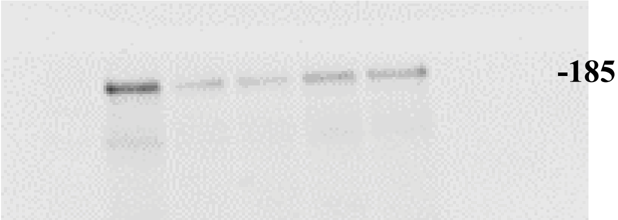

-185

Fig.5: OE33 cells

HER2

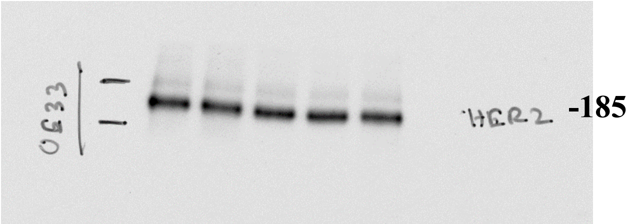

-185

Fig.5: OE33 cells

Actin

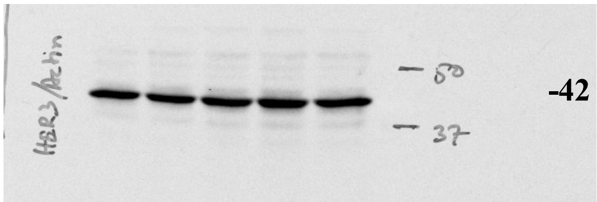

-42

Full-blots for Figure 5

Fig.5: KYSE-410 cells

P-HER3 (pY1289)

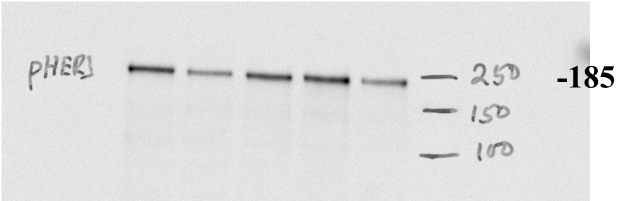

-185

Fig.5: KYSE-410 cells

P-HER3 (pY1197)

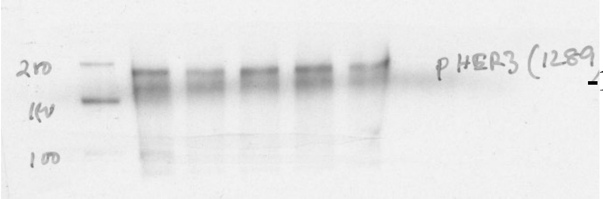

-185

Fig.5: KYSE-410 cells

P-HER3 (pY1222)

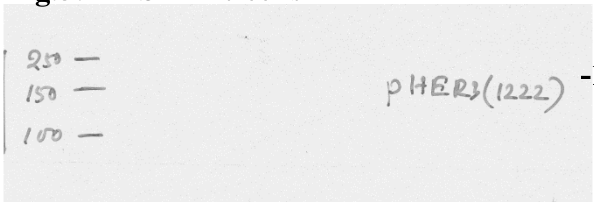

-185

Fig.5: KYSE-410 cells

HER3

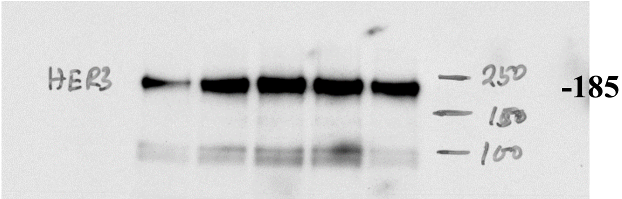

-185

Fig.5: KYSE-410 cells

P-HER2 (pY1248)

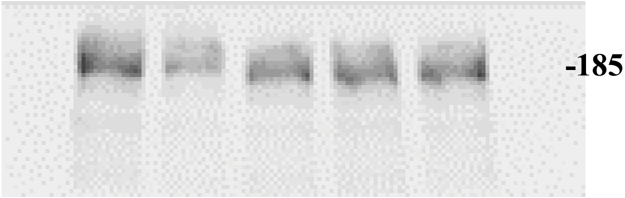

-185

Fig.5: KYSE-410 cells

HER2

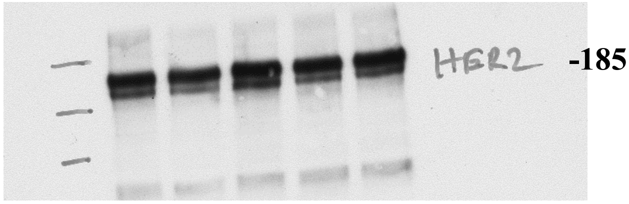

-185

Fig.5: KYSE-410 cells

Actin

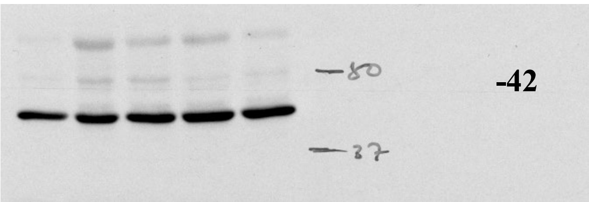

-42

Fig.5: OE21 cells

P-HER3 (pY1289)

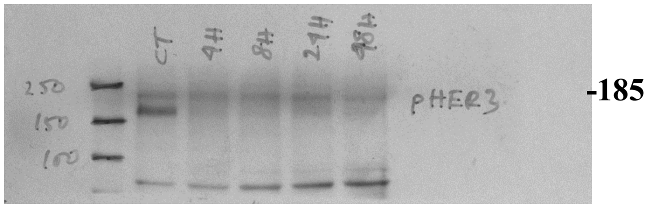

-185

Fig.5: OE21 cells

P-HER3 (pY1197)

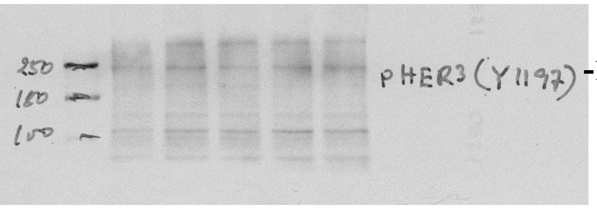

-185

Fig.5: OE21 cells

P-HER3 (pY1222)

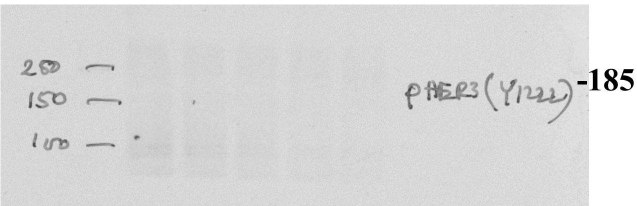

-185

Fig.5: OE21 cells

HER3

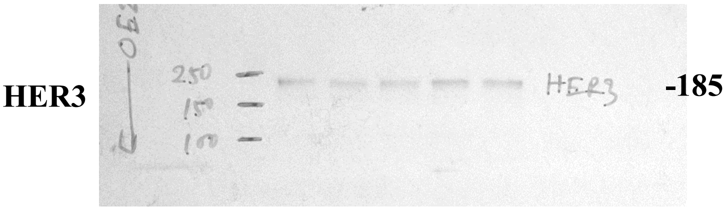

-185

Fig.5: OE21 cells

P-HER2 (pY1248)

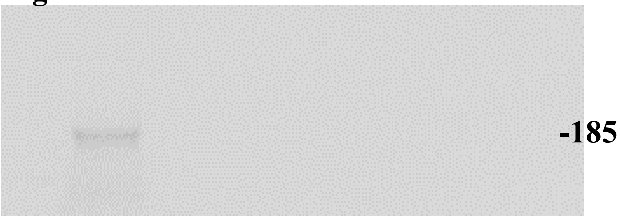

-185

Fig.5: OE21 cells

HER2

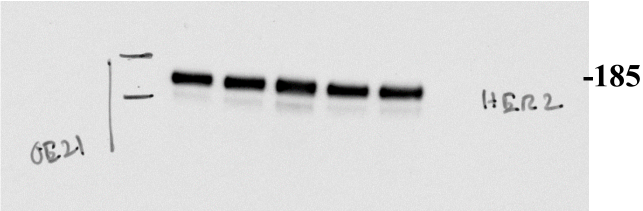

-185

Fig.5: OE21 cells

Actin

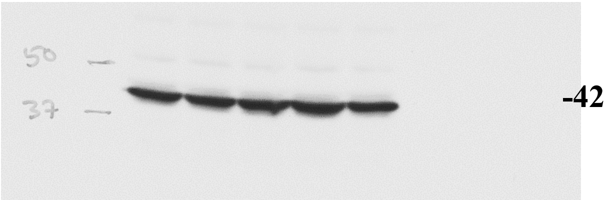

-42

Full-blots for Figure 5

Fig.5: TE7 cells

P-HER3 (pY1289)

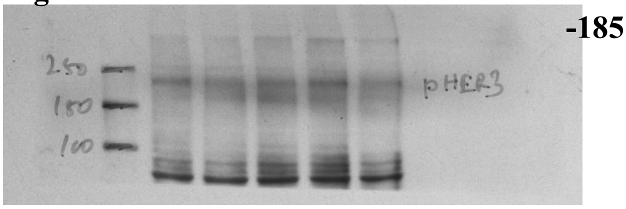

-185

Fig.5: TE7 cells

P-HER3 (pY1197)

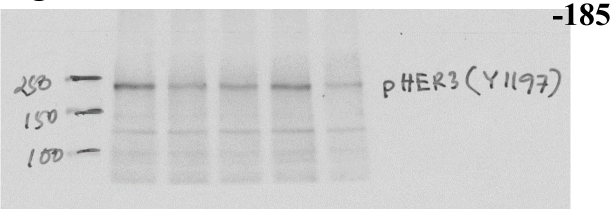

-185

Fig.5: TE7 cells

P-HER3 (pY1222)

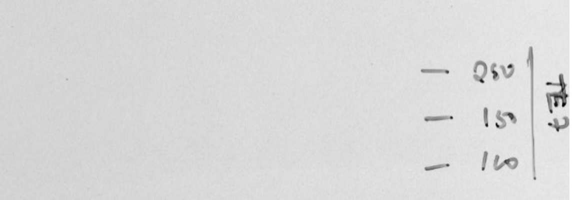

-185

Fig.5: TE7 cells

HER3

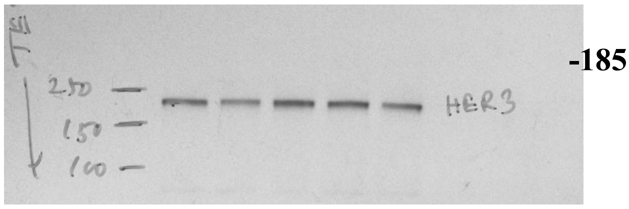

-185

Fig.5: TE7 cells

P-HER2 (pY1248)

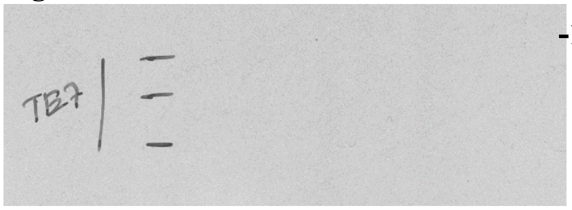

-185

Fig.5: TE7 cells

HER2

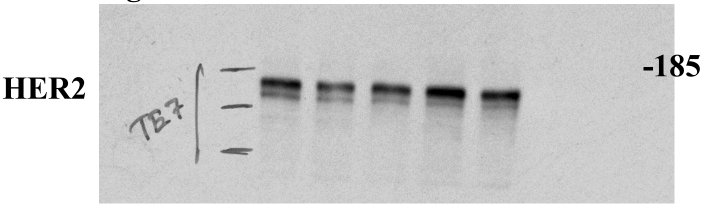

-185

Fig.5: TE7 cells

Actin

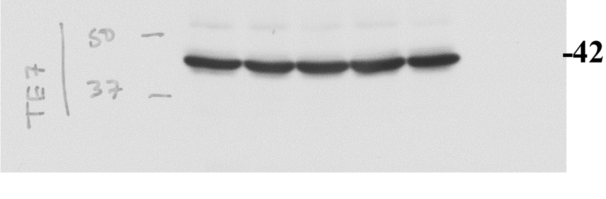

-42

Fig.5: Calu 3 cells

P-HER3 (pY1289)

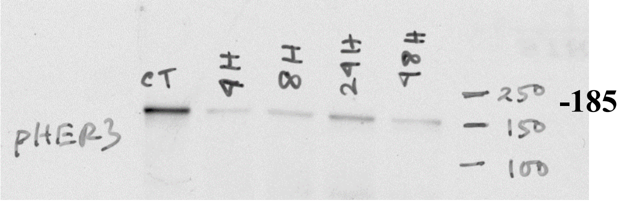

-185

Fig.5: Calu 3 cells

P-HER3 (pY1197)

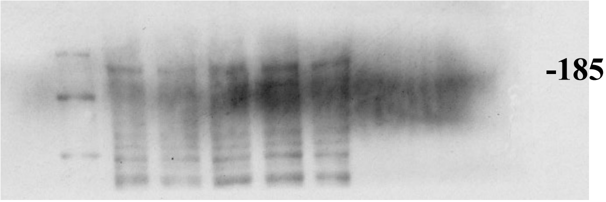

-185

Fig.5: Calu 3 cells

P-HER3 (pY1222)

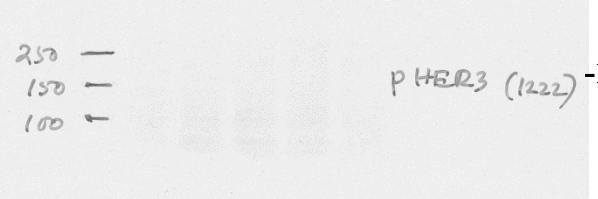

-185

Fig.5: Calu 3 cells

HER3

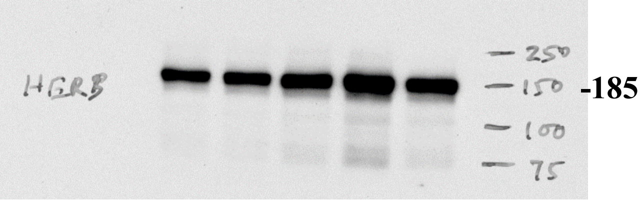

-185

Fig.5: Calu 3 cells

P-HER2 (pY1248)

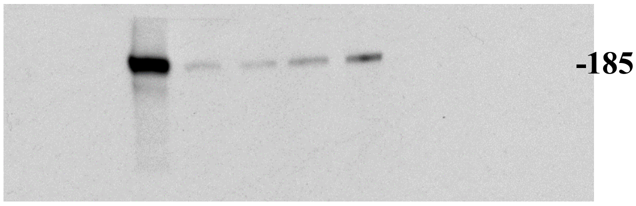

-185

HER2

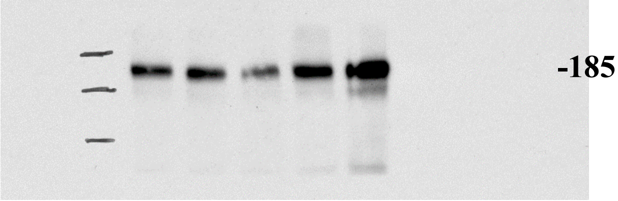

-185

Fig.5: Calu 3 cells

Actin

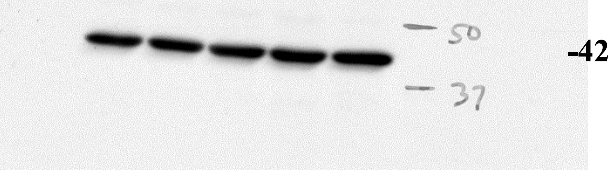

-42

Full-blots for Figure 5

Fig.5: VMRCLCP cells

P-HER3 (pY1289)

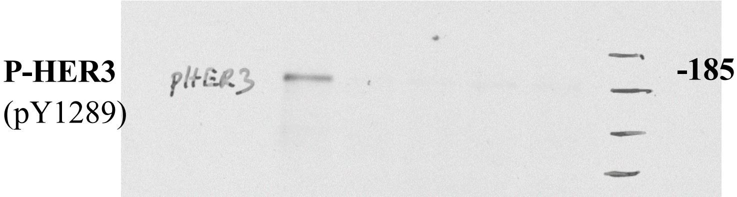

-185

Fig.5: VMRCLCP cells

P-HER3 (pY1197)

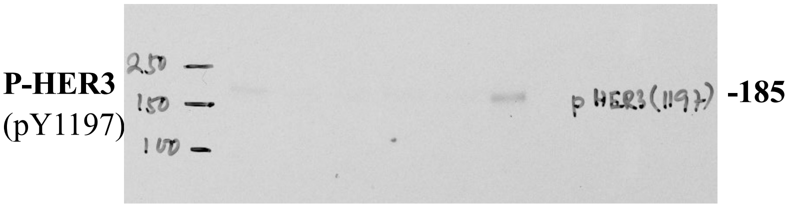

-185

Fig.5: VMRCLCP cells

P-HER3 (pY1222)

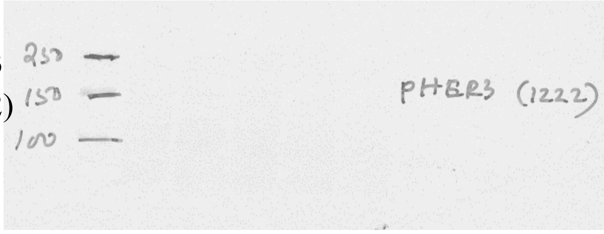

-185

Fig.5: VMRCLCP cells

HER3

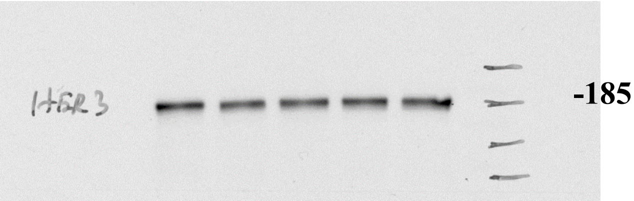

-185

Fig.5: VMRCLCP cells

P-HER2 (pY1248)

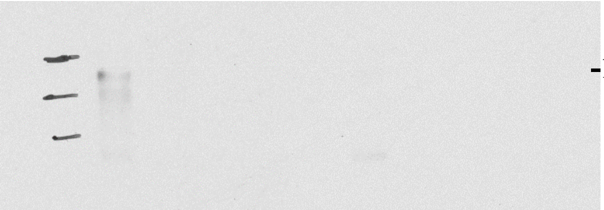

-185

Fig.5: VMRCLCP cells

HER2

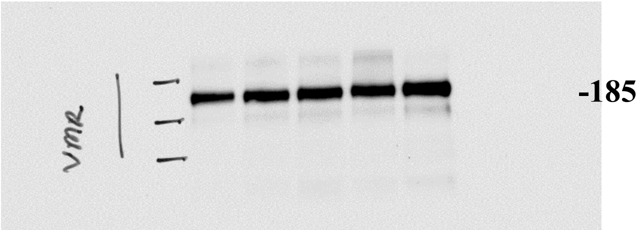

-185

Fig.5: VMRCLCP cells

Actin

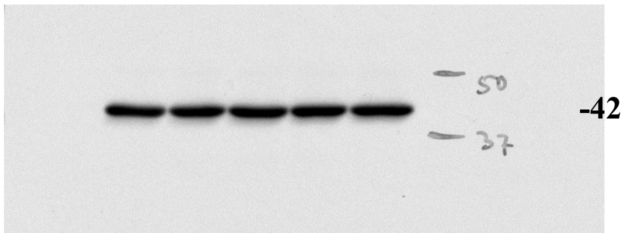

-42

Fig.5: H2170 cells

P-HER3 (pY1289)

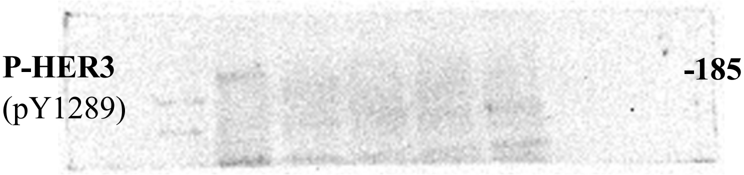

-185

Fig.5: H2170 cells

P-HER3 (pY1197)

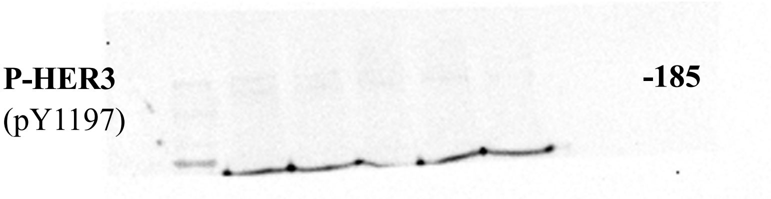

-185

Fig.5: H2170 cells

P-HER3 (pY1222)

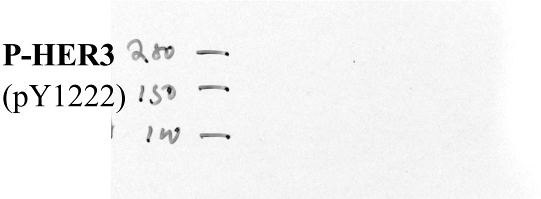

-185

Fig.5: H2170 cells

HER3

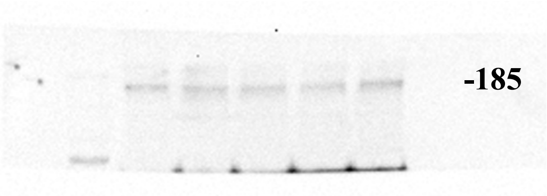

-185

Fig.5: H2170 cells

P-HER2 (pY1248)

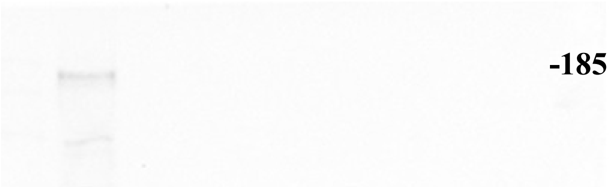

-185

Fig.5: H2170 cells

HER2

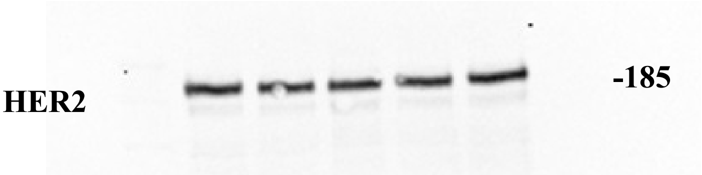

-185

Fig.5: H2170 cells

Actin

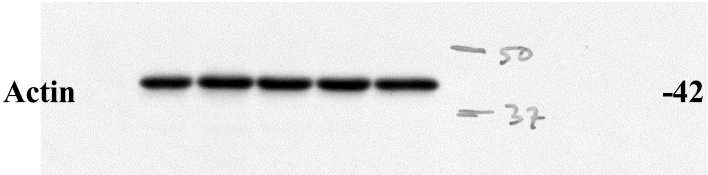

-42

Full-blots for Figure 5

Fig.5: BICR16 cells

P-HER3 (pY1289)

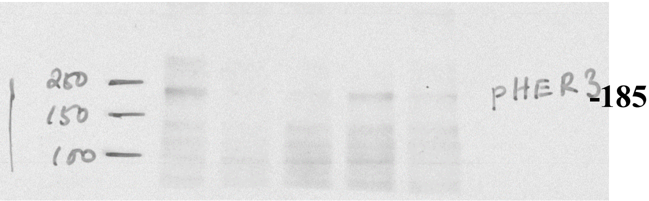

-185

Fig.5: BICR16 cells

P-HER3 (pY1197)

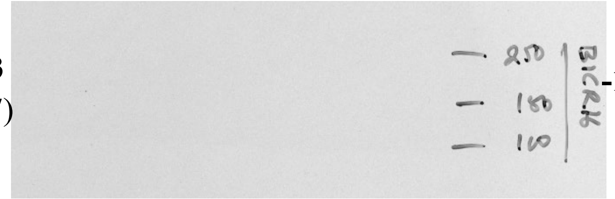

-185

Fig.5: BICR16 cells

P-HER3 (pY1222)

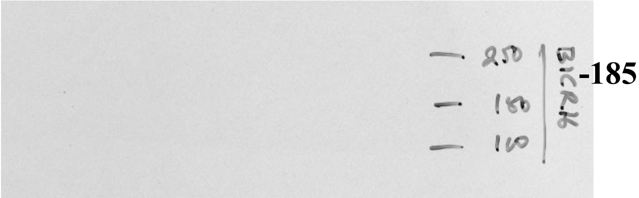

-185

Fig.5: BICR16 cells

HER3

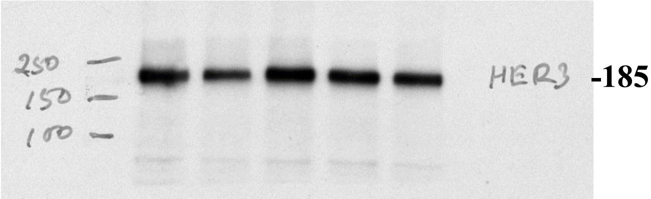

-185

Fig.5: BICR16 cells

P-HER2 (pY1248)

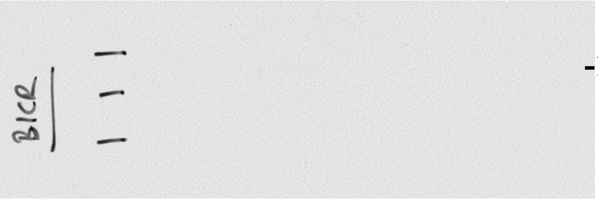

-185

Fig.5: BICR16 cells

HER2

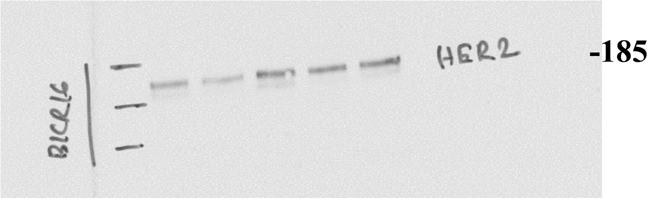

-185

Fig.5: BICR16 cells

Actin

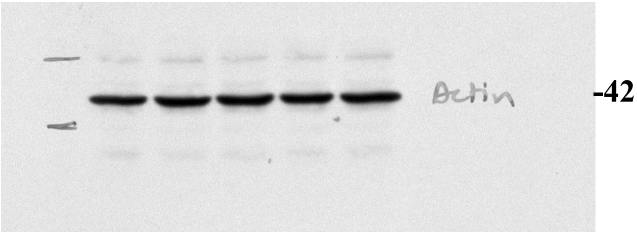

-42

Fig.5: Cal27 cells

P-HER3 (pY1289)

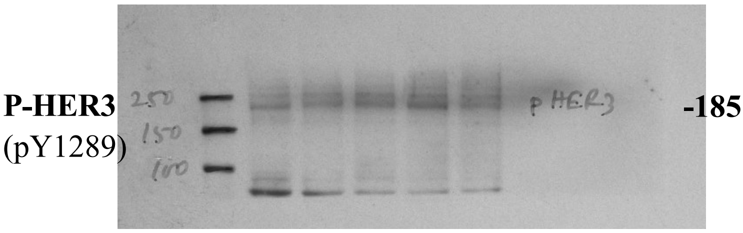

-185

Fig.5: Cal27 cells

P-HER3 (pY1197)

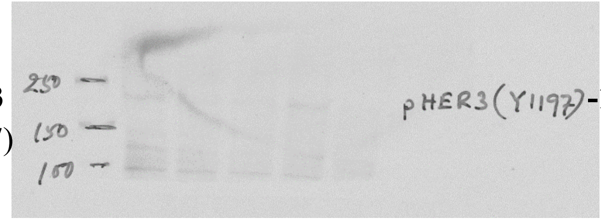

-185

Fig.5: Cal27 cells

P-HER3 (pY1222)

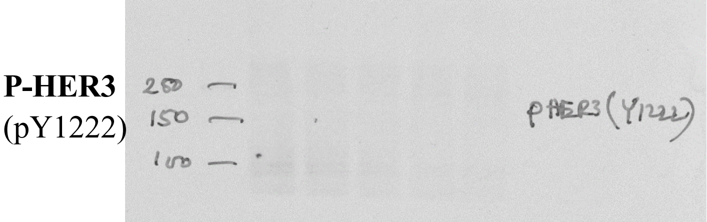

-185

Fig.5: Cal27 cells

HER3

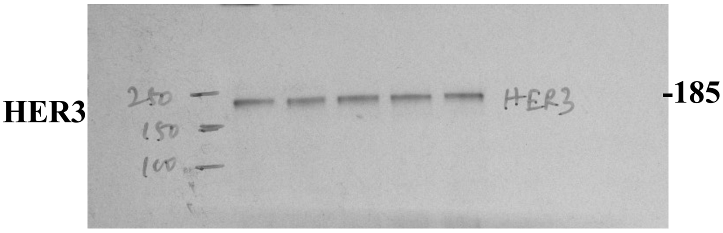

-185

Fig.5: Cal27 cells

P-HER2 (pY1248)

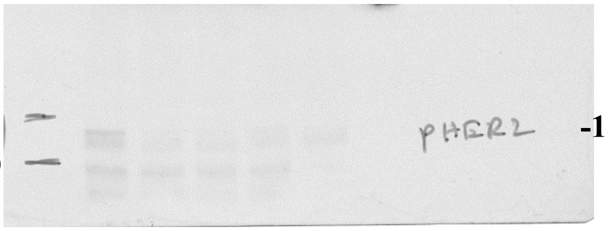

-185

Fig.5: Cal27 cells

HER2

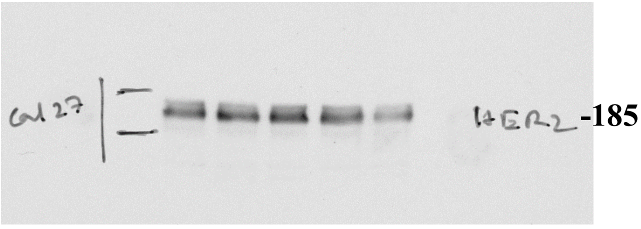

-185

Fig.5: Cal27 cells

Actin

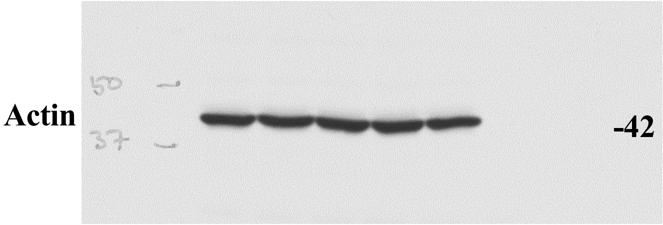

-42

<u>Full-blots for Figure 5</u>

Fig.5: TEN cells

P-HER3 (pY1289)

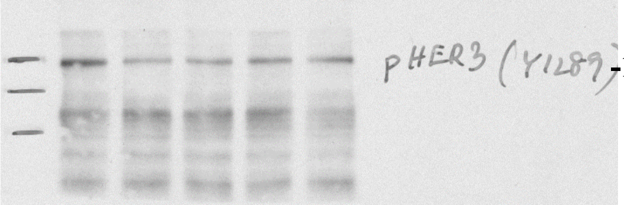

-185

Fig.5: TEN cells

P-HER3 (pY1197)

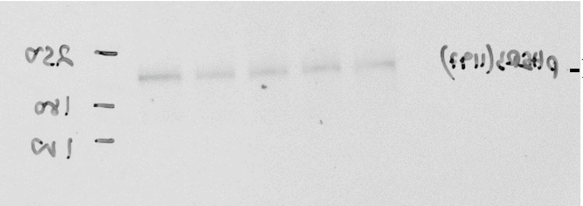

-185

Fig.5: TEN cells

P-HER3 (pY1222)

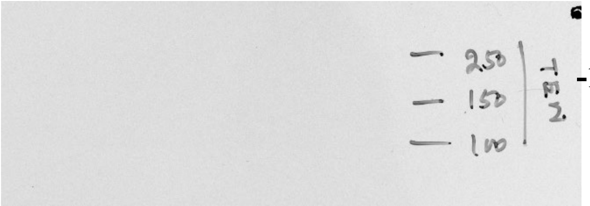

-185

Fig.5: TEN cells

HER3

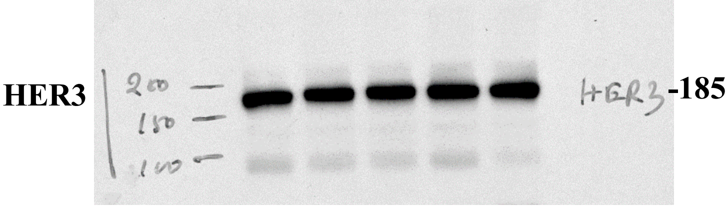

-185

Fig.5: TEN cells

P-HER2 (pY1248)

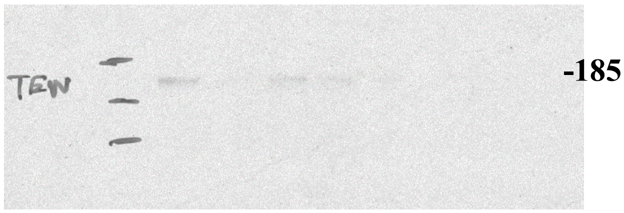

-185

Fig.5: TEN cells

HER2

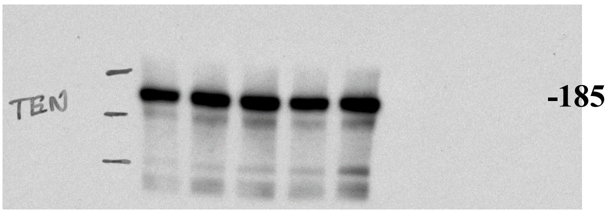

-185

Fig.5: TEN cells

Actin

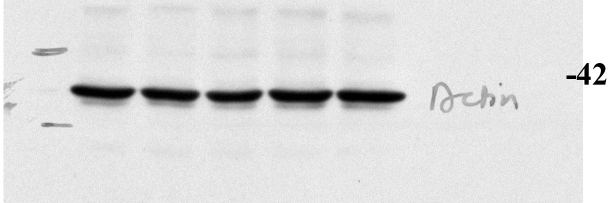

-42

Fig.5: USPC-ARC1 cells

P-HER3 (pY1289)

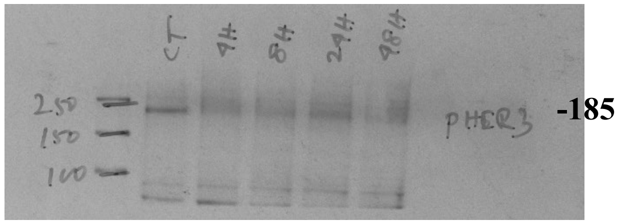

-185

Fig.5: USPC-ARC1 cells

P-HER3 (pY1197)

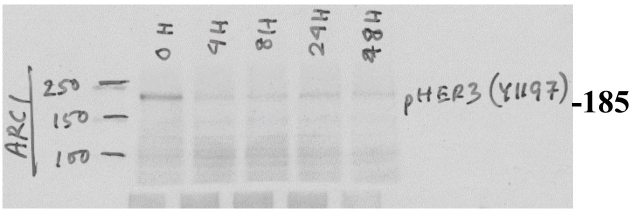

-185

Fig.5: USPC-ARC1 cells

P-HER3 (pY1222)

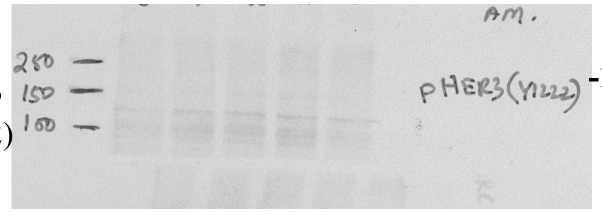

-185

Fig.5: USPC-ARC1 cells

HER3

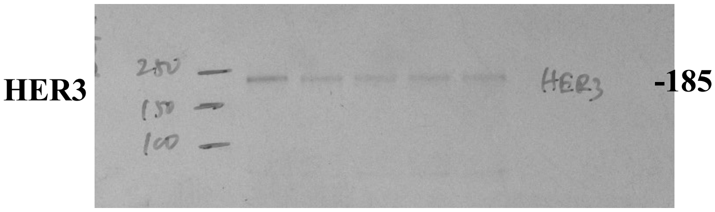

-185

Fig.5: USPC-ARC1 cells

P-HER2 (pY1248)

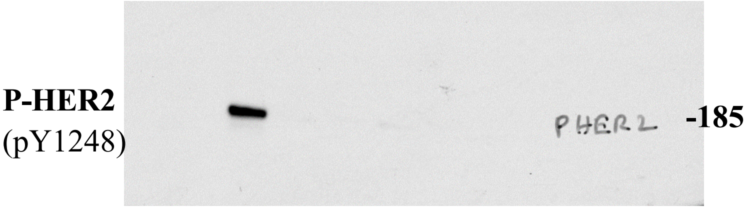

-185

Fig.5: USPC-ARC1 cells

HER2

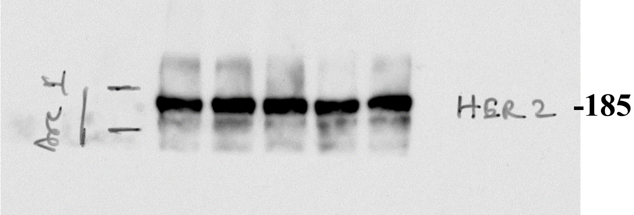

-185

Fig.5: USPC-ARC1 cells

Actin

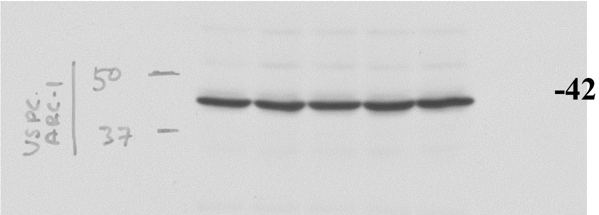

-42

Full-blots for Figure 5

Fig.5: USPC-ARC2 cells

P-HER3 (pY1289)

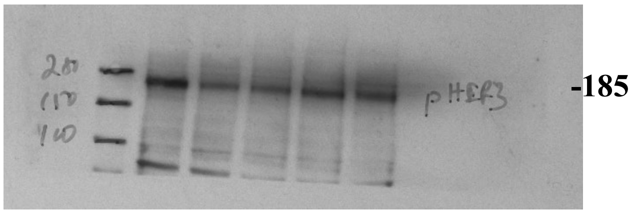

-185

Fig.5: USPC-ARC2 cells

P-HER3 (pY1197)

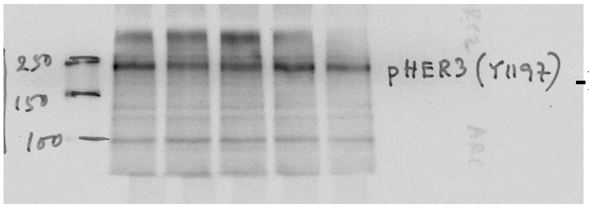

-185

Fig.5: USPC-ARC2 cells

P-HER3 (pY1222)

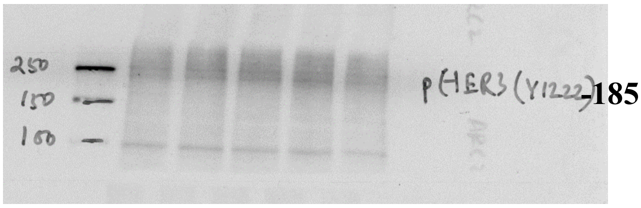

-185

Fig.5: USPC-ARC2 cells

HER3

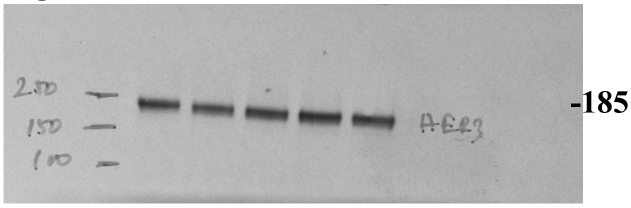

-185

Fig.5: USPC-ARC2 cells

P-HER2 (pY1248)

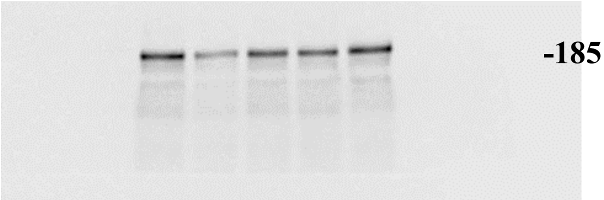

-185

Fig.5: USPC-ARC2 cells

HER2

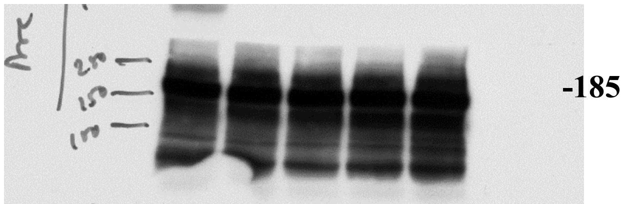

-185

Fig.5: USPC-ARC2 cells

Actin

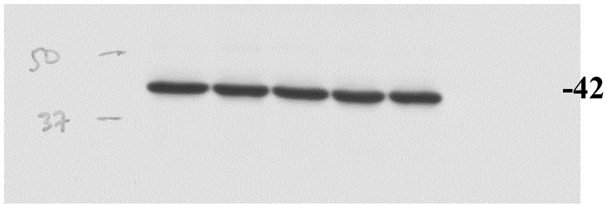

-42

Fig.5: USPC-ARC4 cells

P-HER3 (pY1289)

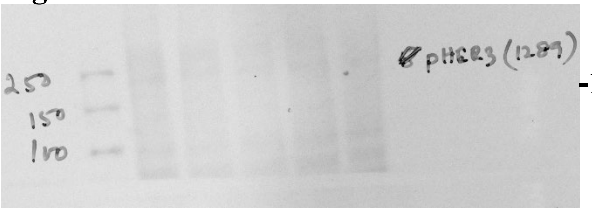

-185

Fig.5: USPC-ARC4 cells

P-HER3 (pY1197)

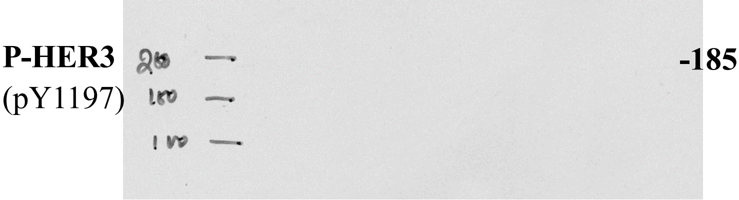

-185

Fig.5: USPC-ARC4 cells

P-HER3 (pY1222)

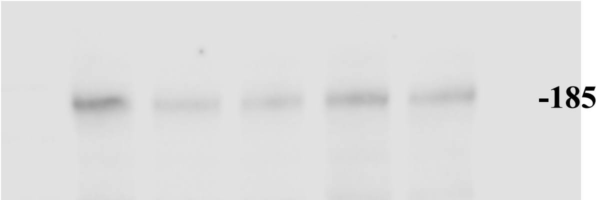

-185

Fig.5: USPC-ARC4 cells

HER3

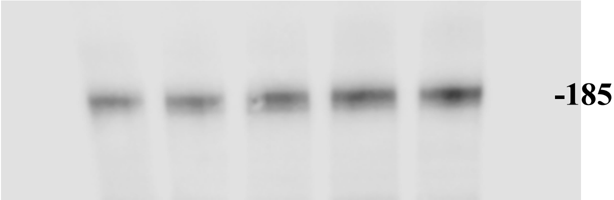

-185

Fig.5: USPC-ARC4 cellsc

P-HER2 (pY1248)

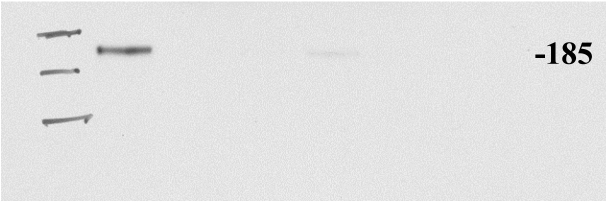

-185

Fig.5: USPC-ARC4 cells

HER2

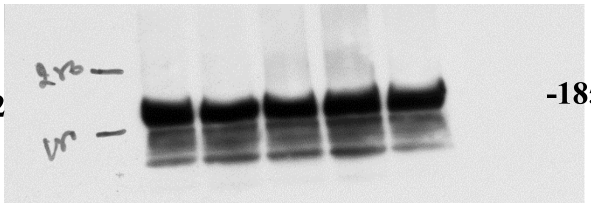

-185

Fig.5: USPC-ARC4 cells

Actin

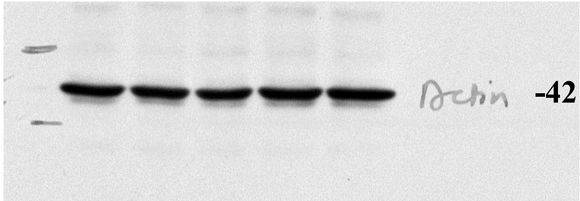

-42

Full blots for Figure 6

Fig. 6A: HER3

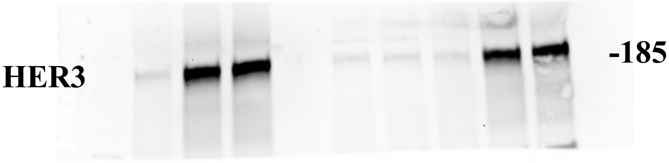

Fig. 6A: Actin

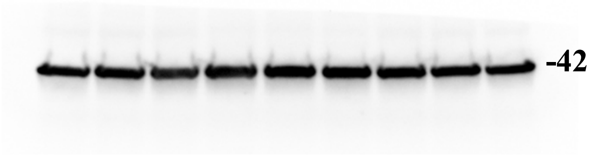

Fig. 6E: OE19 Tumors, HER3 blot

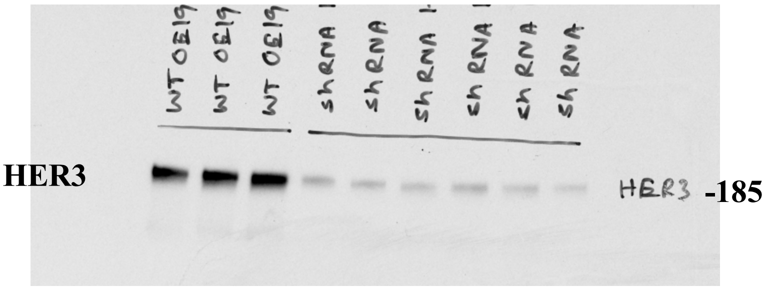

Fig. 6E: OE Tumors, Actin blot

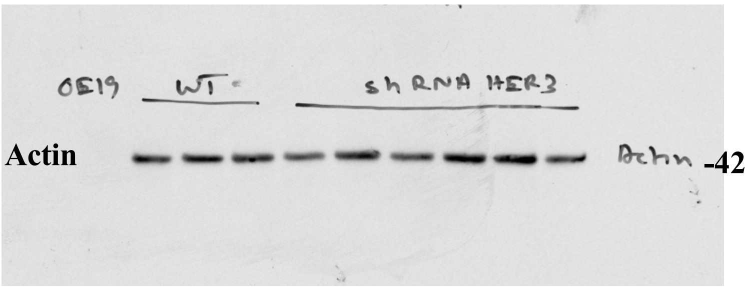

Fig. 6E: H2170 Tumors, HER3 blot

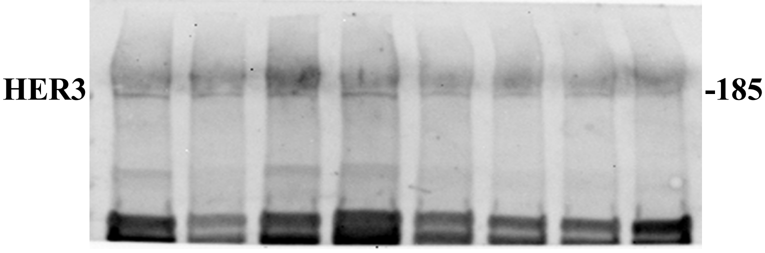

Fig. 6E: H2170 Tumors, Actin blot

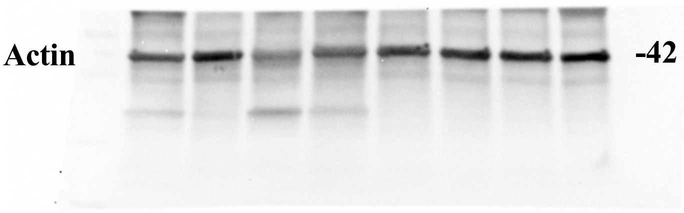

Fig. 6E: N87 Tumors, HER3 blot

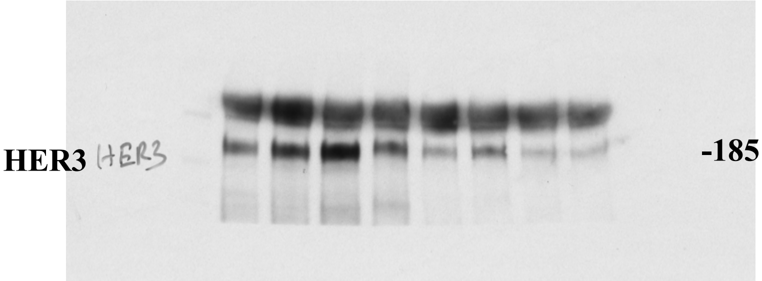

Fig. 6E: N87 Tumors, Actin blot

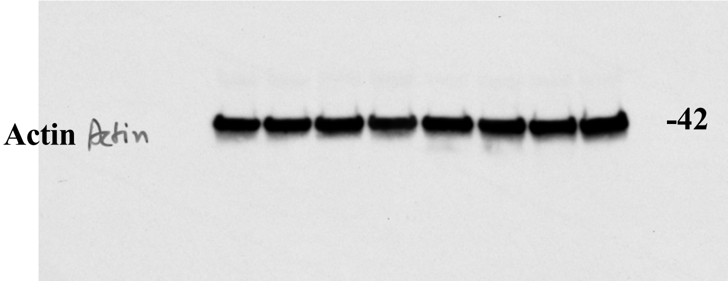

Supplement: Supplementary file 1 — Supplementary Information [file 41598_2021_88683_MOESM1_ESM.pdf]
